# RdJ detection tests to identify a unique MRSA clone of ST105-SCC*mec*II lineage and its variants disseminated in the metropolitan region of Rio de Janeiro

**DOI:** 10.3389/fmicb.2023.1275918

**Published:** 2023-11-20

**Authors:** Matheus Assis Côrtes Esteves, Alice Slotfeldt Viana, Gabriela Nogueira Viçosa, Ana Maria Nunes Botelho, Ahmed M. Moustafa, Felipe Raposo Passos Mansoldo, Adriana Lucia Pires Ferreira, Alane Beatriz Vermelho, Bernadete Teixeira Ferreira-Carvalho, Paul Joseph Planet, Agnes Marie Sá Figueiredo

**Affiliations:** ^1^Departamento de Microbiologia Médica, Universidade Federal do Rio de Janeiro, Rio de Janeiro, Brazil; ^2^Biomanguinhos, Fundação Oswaldo Cruz, Rio de Janeiro, Brazil; ^3^Children’s Hospital of Philadelphia, Philadelphia, PA, United States; ^4^Department of Pediatrics, University of Pennsylvania, Philadelphia, PA, United States; ^5^Departamento de Microbiologia Geral, Universidade Federal do Rio de Janeiro, Rio de Janeiro, Brazil; ^6^Hospital Universitário Clementino Chagas Filho, Universidade Federal do Rio de Janeiro, Rio de Janeiro, Brazil; ^7^Dasa Medicina Diagnóstica, Duque de Caxias, Brazil; ^8^Faculdade de Medicina, Programa de Pós-graduação em Patologia, Universidade Federal Fluminense, Niterói, Brazil

**Keywords:** emerging MRSA, rapid test for clonality detection, biomarker search, pangenome matrix, bacterial genomics

## Abstract

Hospital bloodstream infection (BSI) caused by methicillin-resistant *Staphylococcus aureus* (MRSA) is a major cause of morbidity and mortality and is frequently related to invasive procedures and medically complex patients. An important feature of MRSA is the clonal structure of its population. Specific MRSA clones may differ in their pathogenic, epidemiological, and antimicrobial resistance profiles. Whole-genome sequencing is currently the most robust and discriminatory technique for tracking hypervirulent/well-adapted MRSA clones. However, it remains an expensive and time-consuming technique that requires specialized personnel. In this work, we describe a pangenome protocol, based on binary matrix (1,0) of open reading frames (ORFs), that can be used to quickly find diagnostic, apomorphic sequence mutations that can serve as biomarkers. We use this technique to create a diagnostic screen for MRSA isolates circulating in the Rio de Janeiro metropolitan area, the RdJ clone, which is prevalent in BSI. The method described here has 100% specificity and sensitivity, eliminating the need to use genomic sequencing for clonal identification. The protocol used is relatively simple and all the steps, formulas and commands used are described in this work, such that this strategy can also be used to identify other MRSA clones and even clones from other bacterial species.

## 1 Introduction

*Staphylococcus aureus* is a leading cause of bloodstream infection (BSI) in hospitals and the community, which has an associated mortality rate of 20% to 30% (Kwiecinski and Horswill, 2020). The risk associated with patients over 60 years of age is even greater, with lethality reaching more than half of those infected (Loftus et al., 2022). Furthermore, BSI caused by methicillin-resistant *S. aureus* (MRSA) is recognized as an important health problem, as it appears to increase morbidity and mortality compared to those associated to methicillin-susceptible *S. aureus* (MSSA) strains (Kwiecinski and Horswill, 2020). Reported percentages of MRSA in BSI in Latin-American countries are in the alarming range of 40% to 60% of all *S. aureus* BSIs (Arias et al., 2017; Iuliano et al., 2018). An important strategy to prevent the spread of multidrug-resistant (MDR) bacteria, especially those circulating in healthcare environments, is the rapid identification and control of pathogens. High-income countries that have invested in a “search-and-destroy” policy for MDR pathogens have very low rates of MRSA in their hospitals (Souverein et al., 2016). Whole Genome Sequencing (WGS) has proven useful to accurately identify clones, track clonal transmission, and limit bacterial outbreaks caused by MDR bacteria (Yang et al., 2006; Lakhundi and Zhang, 2018; Beukers et al., 2020). The importance of identifying MRSA clones in hospitals also lies in the fact that different MRSA clones may be involved in different clinical and epidemiological scenarios and may also have different virulence and MDR profiles (Côrtes et al., 2021; Viana et al., 2021). However, WGS is a time-consuming technique that requires specialized knowledge, the use of bioinformatics programs to determine clonality with reliable power, and expensive resources that may not be available in all settings.

Recently, a unique MRSA clone, the Rio de Janeiro (RdJ) strain, of the lineage ST105 (CC5)-SCC*mec*II-002 and its variants emerged in several hospitals located in the metropolitan region of Rio de Janeiro city (Viana et al., 2021). Notably, RdJ was found more frequently in blood samples and exhibited a superior ability to evade THP-1 monocyte killing compared to its clonal complex 5 (CC5) close relatives (Viana et al., 2021). However, due to the high genetic similarity between RdJ and other CC5 strains, until now, the only approach available to accurately identify RdJ is WGS. In this work, to design a simple RdJ detection test, we created a binary matrix (1,0) that compares two groups of pangenome open reading frames (ORFs; RdJ and non-RdJ) based on presence/absence of ORFs with 100% nucleotide identity. We used this matrix to identify apomorphic mutations and find specific biomarkers to identify members of the RdJ clone.

## 2 Materials and methods

### 2.1 Development of RdJ detection tests

#### 2.1.1 Construction of pangenome matrix

A total of 661 MRSA genomes from clonal complex 5 (CC5) were used. These genomes included MRSA strains (*N* = 179) isolated from patients hospitalized in Rio de Janeiro metropolitan region that were previously sequenced by our group (Viana et al., 2021); in addition to CC5 genomes available in the GenBank (*N* = 482). The FASTA files of these 661 CC5 genomes are accessible by the Mendeley Data Repository (doi: 10.17632/jd5tsjp4g6.1). A concatenation of all open reading frames (ORFs) from the genome sequence of strain N315 (ST5-SCC*mec*II; Acc: BA000018.3) was used as query to conduct nBlast using pangenome groups of CC5-RdJ and CC5-non-RdJ as the subject. A binary pangenomic matrix (1,0) was constructed based on presence/absence of ORFs with 100% nucleotide identity for each pangenomic group. Student’s *t*-test (two tailed for independent groups) was used to calculate differences (presence/absence) for each ORF between groups. A volcano scatterplot was defined with the Log_2_fold change and−Log*p* value (x,y coordinates; respectively).

The ORF sequences with the lowest *p-*values were localized in the genomes of the Brazilian strain CD16-016 (RdJ strain; Acc: GCA_021010535.1) using the “map to reference tool” (Geneious Prime software; https://www.geneious.com) and curated manually using NCBI^1^ or Uniprot-SwissProt^2^ databases. The Microsoft Excel^3^ program formulas and commands used for these analyses are available at Mendeley Data Repository (doi: 10.17632/jd5tsjp4g6.1). Genotyping (MLST, SCC*mec* typing, and *spa* typing) was predicted for all strains analyzed from next-generation sequencing data using the Center for Genomic Epidemiology platform^4^ with the software Multi Locus Sequence Typing (MLST 2.0), SCCmecFinder and spaTyper, respectively.

#### 2.1.2 Selection of the biomarker for the development of RdJ detection tests

The distribution of the volcano plot revealed an apomorphic single nucleotide mutation (A1106G) in the *aur* gene, encoding the protease aureolysin, that differentiates members of the RdJ clade from their ancestors and close relatives. To further investigate the exclusivity of this mutation for the ST105 RdJ isolates, the coding sequences (CDS) of the total 71,017 annotated genomes of *S. aureus* were downloaded from the NCBI GenBank on April 23, 2023, using the NCBI dataset tool, and Unix xargs command was used to combine find and grep commands to search for the 20 nucleotides (TATAAGGGCCAGTCAGGCGC) in all genomes. This sequence corresponds to the location 1,099–1,118 nt of the *aur* gene, comprising the *aur* mutation (A1106G), in the genome of the RdJ strain CD16-016 (Acc: GCA_021010535.1). In addition, the sequence types (ST) of all 71,071 *S. aureus* genomes were determined using MLST tool.^5^ The *aur* gene sequence of all RdJ genomes was analyzed using Geneious Prime platform for mutations that could lead to creation or deletion of endonuclease restriction sites. Analysis of the *aur* mutation (A1106G), unique to RdJ isolates, showed that it creates a new and unique restriction site for the *Bgl*I endonuclease. This feature was exploited for the design of RdJ detection tests.

#### 2.1.3 Phylogenetic tree

A maximum likelihood tree was constructed using 180 methicillin-resistant CC5 genomes from hospitals located in the metropolitan region of Rio de Janeiro city, Brazil, in the period 2014–2017, along with 542 genomes obtained from GenBank and SRA (Supplementary Table S1). Single nucleotide polymorphism (SNP) alignment was performed using Realphy 1.13 pipeline.^6^ The unrooted tree was built using Geneious Tree Builder^7^ with Tamura-Nei distance model and Neighbor-Joining method. The tree was visualized using iTol v6.^8^ We used this tree study to highlight the RdJ clade carrying the apormorphic SNP mutation (A1106G) in *aur* gene.

#### 2.1.4 Oligonucleotide design

Our strategy was to design a method capable of (i) confirming the presence of *mecA* gene, (ii) detecting the clonal complex 5 (CC5) by the Agr type II (*agrII*; specific for CC5 strains), and (iii) identifying the RdJ isolates by detecting the *aur* mutation A1106G. Primers were designed for a multiplex PCR (mPCR) to amplify segments of *mecA*, *agr*II, and *aur* genes. Subsequently, the mPCR product was digested with the endonuclease *Bgl*I and the restriction fragment length polymorphisms (RFLP) analyzed. Table 1 shows the list of primers used for mPCR-RFLP method designed for the RdJ detection tests.

**Table 1:** Primers used for RdJ detection tests.

Primer	Direction	Sequence (5′–3′)	Amplicon (bp)	References
MecA	Forward	AGATGATACCTTCGTTCCACT	585	This study
	Reverse	CTGGTGAAGTTGTAATCTGGA		
AgrII	Forward	ATGTGTGCTCATGCAAAGTCTT	160	Beltrame et al. (2012)
	Reverse	CATGTGCATAAATAACAACGG		
Aur	Forward	ACTTGGCGATACAAAAGATATCA	752	This study
	Reverse	ATTCCGTTAATGCTCGGTAGT		

### 2.2 Detailed RdJ detection tests protocol

DNA was prepared using the Wizard Genomic DNA Purification Kit (Cat# A1125; Promega Corporation, Madison, WI, United States) following the manufacturer’s protocol, except that a lysis step with 200 U lysostaphin (Cat# L7386; Sigma-Aldrich Brasil Ltda; São Paulo, SP, Brazil) in 500 μL of the bacterial suspension in 50 mM EDTA pH 8.0 was used to replace the enzymatic lysis suggested by the manufacturer.

The mPCR reaction was prepared using 300–400 ng DNA, 0.2 μM of each primer (forward and reverse) MecA and Aur, and 0.4 μM of AgrII primer. GoTaq Colorless Master Mix (Cat# M7133; Promega) was used as suggested by the manufacturer. Finally, the reaction was supplemented with dNTP Mix (Cat#U1515; Promega) to a final concentration of 0.4 mM in 25 μL of final volume.

The PCR program was the following: initial denaturation at 95°C for 5 min; followed by 30 cycles of denaturation at 94°C for 45 s, annealing at 52°C for 45 s and extension at 72°C for 40s; with final extension at 72°C for 7 min. Amplification was performed on a Veriti™ 96-Well ThermalCycler thermal cycler (Cat#4452299; Applied Biosystems; Foster City, CA, United States).

The mPCR product was treated with 10 U of *Bgl*I and the reaction performed as recommended by the manufacturer (Cat# R0143L; New England Biolabs, Ipswich, MA, United States). After incubation at 37°C/1 h, 1 μL of 6 X Gel Loading Dye (Cat#P1011-1; Sinapse Inc., Brazil) was added to an aliquot of 5 μL of the fragmented product and applied into the slot of a 2% agarose gel in 1 X TAE (40 mM Tris-acetate, 10 mM EDTA, pH 8.3). The DNA gel electrophoresis was performed at 120 V for 50 min. Gels were treated for 20 min with 0.5 μg/mL ethidium bromide (Cat#E7637OBS; Sigma Laboratories, Saint Louis, MO, United States) and visualized on a Gel Doc EZ System with UV light (Cat#1708270, Bio-Rad, Hercules, CA, United States).

### 2.3 Evaluation of RdJ detection tests

#### 2.3.1 Bacterial collection

A collection of 217 *S. aureus* isolates was analyzed using RdJ detection tests. These isolates were tested in two steps. In the first stage, the researcher responsible for the tests previously knew the WGS data, and in the second stage, the name of each isolate was transformed into a code such that the researcher did not know which isolates were being tested (blind test). For the first stage, a total of 87 isolates were analyzed including MSSA, *N* = 5; non-CC5 MRSA, *N* = 19; ST5(CC5) MRSA, *N* = 33; ST105(CC5) RdJ, *N* = 27; ST105(CC5) non-RdJ, *N* = 3. For the second stage, a total of 130 MRSA isolates were tested including 77 non-RdJ isolates [ST5(CC5), *N* = 53; ST1635(CC5), *N* = 13; ST105(CC5), *N* = 1; and Non-CC5 MRSA, *N* = 10] and 53 RdJ-isolates [ST105(CC5), *N* = 51; and ST4876(CC5), *N* = 2]. All isolates were from our laboratory collection, and were previously genotyped based on MLST, SCC*mec* and *spa* typing using genomic approaches (Viana et al., 2021).

#### 2.3.2 Statistical analysis

The performance of the RdJ detection tests was evaluated using the Diagnostic Test Calculator software^9^ from the University of Illinois, Chicago. Data from genomic and phylogenomic strategies were used as the gold standard for comparison purpose.

## 3 Results

### 3.1 Choosing a biomarker for RdJ detection tests

**Figure 1:**
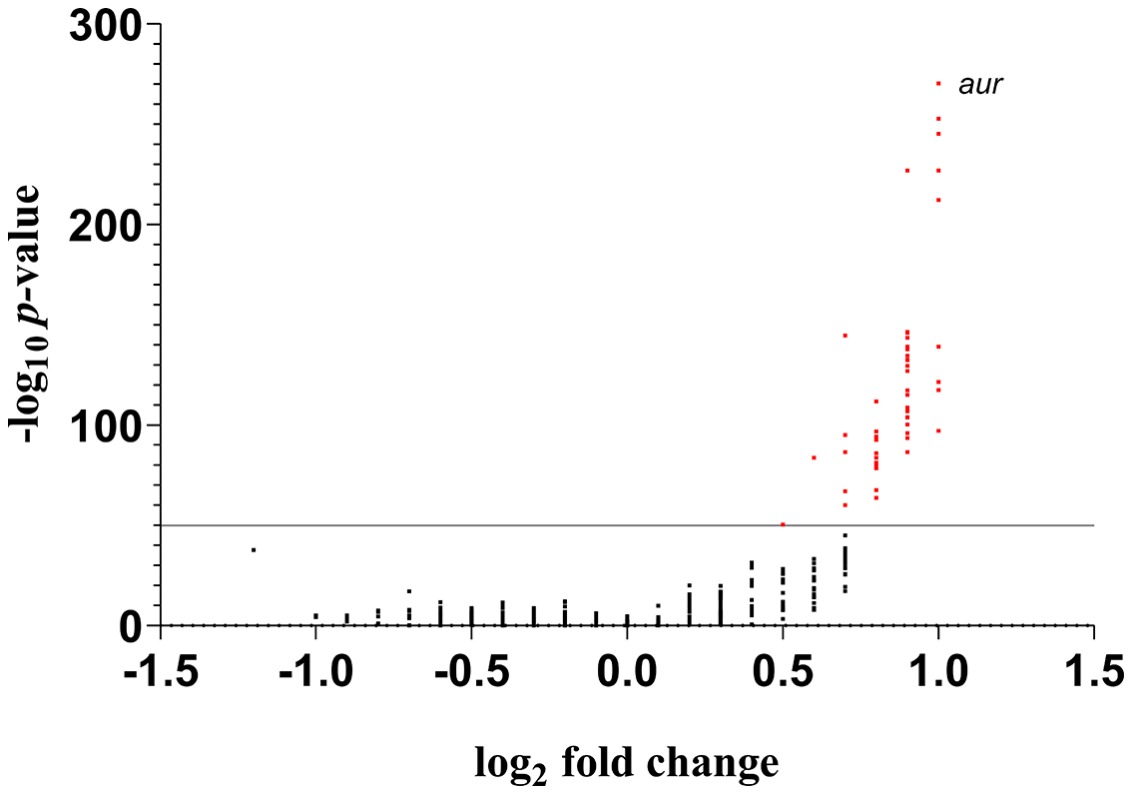
Volcano plot of the distribution of ORFs that differ between the RdJ and all other CC5 MRSA genomes. The red dot represents ORFs whose difference in distributions resulted in *p*-values ≤ 10^−50^.

Using a cutoff of *p* < 10^−50^, the results from the pangenomic matrix revealed 56 ORFs that were found predominantly among the RdJ genomes (Supplementary Table S2). The lowest *value of p* (4.81 × 10^−271^) obtained in this analysis was related to the aureolysin coding sequence (*aur*; Figure 1), indicating that this ORF was a strong candidate to differentiate RdJ from other CC5 MRSA. The zinc metalloproteinase aureolysin has been associated with host immune evasion (Laarman et al., 2011). Five other genes were placed in the Volcano plot in a position close to the *aur* gene, also suggesting their potential to differentiate RdJ from other CC5 MRSA. These coding sequences were also manually analyzed and annotated using RdJ genomes, and include genes for putative products for an anthranilate synthase component, which has a role in amino acid transport/metabolism (Pohl et al., 2009); phosphoenolpyruvate carboxykinase, an enzyme essential for gluconeogenesis that plays a key role in the growth and survival of *S. aureus* cells in the absence of glucose (Harper et al., 2018); a hypothetical protein similar to OppA peptide binding protein, responsible for capturing peptides from the extracellular medium (Hiron et al., 2007); and two hypothetical proteins of unknown function (Table 2).

**Table 2:** Annotation of the six ORFs that showed the highest degree of significance in relation to the ORF differences found between the two CC5 MRSA groups of pangenomes: RdJ and non-RdJ.

ORF product	log2 *fold-change*	−log10 *p*-value	Predicted product	Mutation (nucleotide)	Type of mutation
Aur	1	270.3	Zinc metalloproteinase aureolysin	A1106G	Nonsynonymous
Trpg	1	252.7	Anthranilate synthase component	A337G	Nonsynonymous
Pcka	1	245.3	Phosphoenolpyruvate carboxykinase	A406G	Synonymous
Sa0849	0.9	226.9	Hypothetical protein, similar to peptide binding protein OppA	C1597T	Nonsynonymous
Sa1437	1	226.9	Hypothetical protein	T325C	Synonymous
Sa0667	1	212.3	Hypothetical protein	A406G	Synonymous

**Figure 2:**
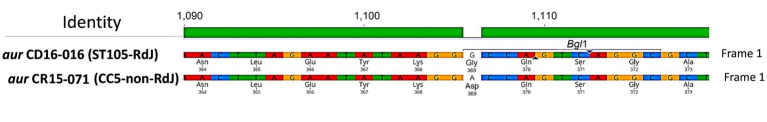
Alignment of nucleotide sequences corresponding to segments of the alleles found in CC5 strains RdJ and non-RdJ comprising the A1106G mutation region. The genomes of MRSA strains CD16-016 (ST105-RdJ, Acc: JAECLS000000000) and MRSA CR15-071 (ST5-SCC*mec*II-t539, non-RdJ, Acc: CP065868) were used to obtain this alignment on Geneious Prime pipeline.

**Figure 3:**
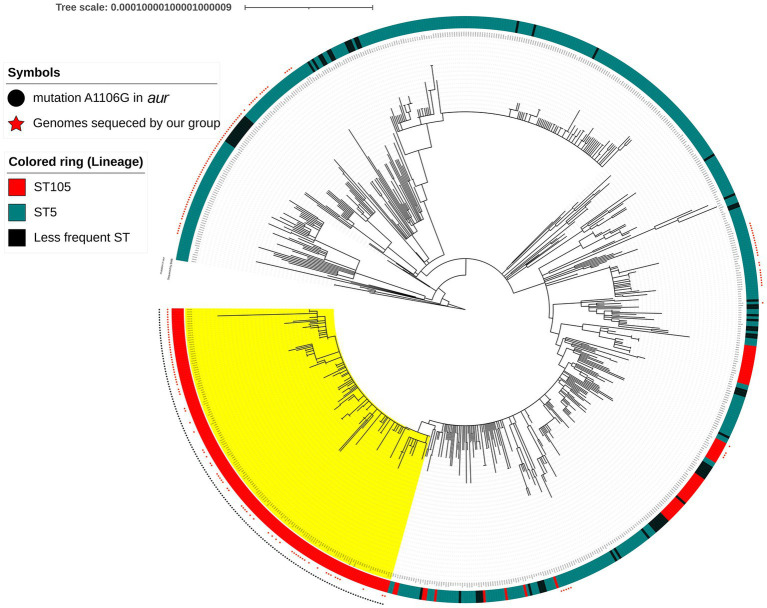
Maximum likelihood phylogenetic tree composed of 180 CC5 genomes from the metropolitan area Rio de Janeiro, sequenced by our group (red star) and 548 CC5 genomes from NCBI databases. The RdJ clade (marked with yellow color) is composed of the ST105(CC5)-SCC*mec*II isolates and few variants: ST4876(CC5)-SCC*mec*II (*n* = 2) and ST5288(CC5)-SCC*mec*II (*n* = 2). Black circle represents the apomorphic mutation (A1106G) in the *aur* gene of the RdJ genomes. The scale indicates substitutions by site.

We analyzed the *aur* mutation (A1106G) to look for possible alteration in an endonuclease site that would allow a simple method for detecting this mutation and found that the A1106G created a *Bgl*I site in RdJ genomes (Figure 2). Search analysis using the 20 nucleotides around the A1106G mutation as a reference against 71,017 *S. aureus* genomes showed that all RdJ genomes (the ones grouped in the RdJ clade; colored in yellow) carried this mutation (Figure 3). Manual curation of the entire *aur* sequence of RdJ genomes showed that only the genomes of strains CD15-152, CD16-151, CD16-152, and UB641 had <100% nucleotide identity (Supplementary Table S1). Each of these strains carries another single-nucleotide mutation, in addition to the expected A1106G, which does not create a *Bgl*I site, and should not interfere with the detection of RdJ isolates.

The analysis of 71,017 *S. aureus* genomes from NCBI database (Supplementary Table S3) revealed a total of 1,385 ST105 sequenced genomes. One hundred and sixty of 71,017 have A1106G mutation in the *aur* gene (Supplementary Table S4). From the 160 genomes, 136 were classified as ST105, 14 show ambiguous results but were considered ST105 by manual curation, and 4 are ST105 variants [ST4876 (*n* = 2) and ST5288 (*n* = 2)] that cluster within RdJ clade (*n* = 154). The remaining six genomes are from different CCs: ST8 (0.02%; *n* = 2/11,198), ST12 (0.79%; *n* = 2/253), ST30 (0.03%; *n* = 1/3,015) and ST479 (4.55%; *n* = 1/22). In the RdJ clade we added six more ST105 RdJ genomes from Rio de Janeiro that could not be downloaded together with the 71,017 *S. aureus* genomes, making a total of 160 RdJ genomes (Supplementary Table S1).

BioSample analysis for the 154 genomes classified as RdJ using NCBI entrez-direct revealed that all were from Brazil, except two from the Netherlands (Supplementary Tables S1, S4). Of the 152 Brazilian genomes, 79 were from Rio de Janeiro and were previously sequenced by our group (Viana et al., 2021). The remaining 73 Brazilian genomes were mostly submitted by the Universidad El Bosque, Colombia and were from MRSA isolates collected from blood in hospitals in the city of São Paulo, where the RdJ clone was predominant among MRSA isolates (53.97%; *n* = 68/126; Arias et al., 2017; Supplementary Table S4). These results confirm that the vast majority of ST105 isolates detected in Brazil (RdJ clone) evolved to acquire a specific *aur* mutation that was conserved during the rapid expansion of this clone in this country.

### 3.2 RdJ detection tests

**Figure 4:**
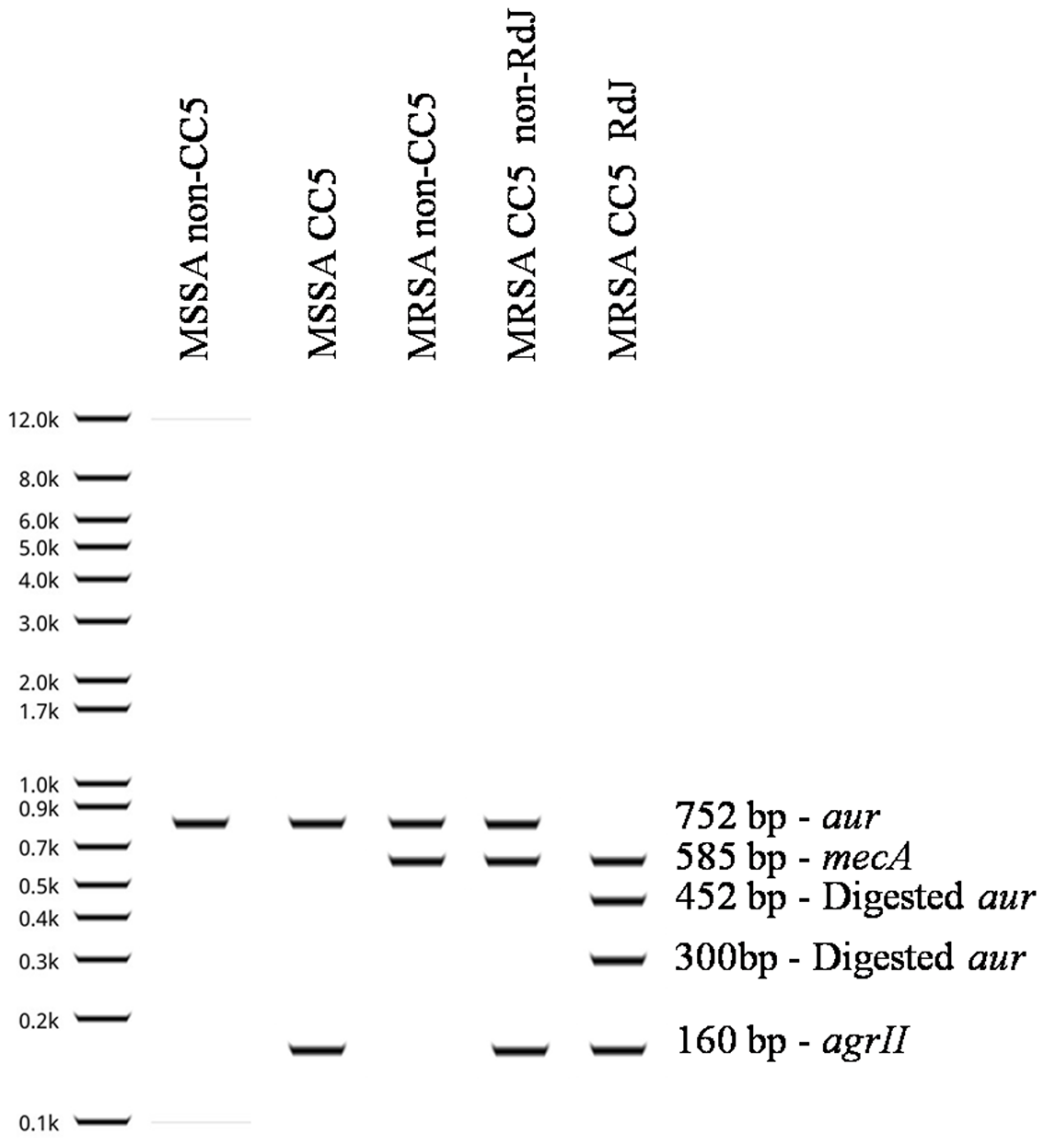
Representation of the RdJ detection tests performed for different types of *Staphylococcus aureus* strains. Shown in this figure are the possible results of the RdJ tests: MSSA non-CC5, MSSA CC5, MRSA non-CC5, MRSA CC5 non-RdJ and MRSA CC5 RdJ. Note that the MRSA CC5 RdJ isolates have two *aur* fragments at 452 bp and 300 bp, whereas MRSA CC5-non-RdJ have wild-type *aur* that lacks a *Bgl*I restriction site. MRSA is distinguished from MSSA by the amplification of a fragment of the *mecA* gene, and CC5 isolates are distinguished from non-CC5 by the *agr*II fragment.

Based on the phylogenomic findings, we designed the multiplex PCR RdJ detection test protocol that is described in detail in the Material & Methods section. This test allows detection of *mecA*, presence/absence of CC5 (predominant clonal complex in Rio de Janeiro), and differentiation of CC5-RdJ from CC5-non-RdJ MRSA isolates (Figure 4).

Once the RdJ detection tests were established, a total of 87 *Staphylococcus* isolates were initially tested. These results are compiled in Supplementary Table S5. All mPCR patterns obtained with RdJ detection tests matched to the expected genotyping and phylogenetic profile found through genomic analyses. After this step, we tested another 130 MRSA isolates in a blind test. Again, 100% of congruence was observed between the results of the RdJ detection tests and genomic phylogeny and genotypes (Supplementary Table S5).

Although the RdJ detection tests were 100% sensitive and 100% specific for the detection of MRSA isolates of the RdJ clone (which mostly belong to the ST105-SCC*mec*II lineage, with exception of two ST4876-SCC*mec*II-t002 isolates), it is important to emphasize that these tests do not have the same sensitivity and specificity for the identification of ST105-SCC*mec*II isolates from Rio de Janeiro that are not in the RdJ clade. Even though the vast majority of RdJ isolates from Rio de Janeiro belong to the ST105-SCC*mec*II lineage, four ST105-SCC*mec*II isolates from Rio de Janeiro (CD15-245, CD15-276, CR15-001, CR14-025) are non-RdJ clade isolates and do not have the A1106G mutation. As expected, these four strains were phylogenetically distinct from the RdJ clone isolates, grouping in another clade along with genomes of isolates from São Paulo, Brazil; Boston, USA; and Venezuela. Furthermore, in the international context, according to our BLAST analysis, non-RdJ ST105-SCC*mec*II do not have the A1106G mutation. The sensitivity, specificity, negative predictive value (NPV) and positive predictive value (PPV) of the RdJ detection tests in relation to their power to identify the ST105-SCC*mec*II lineage from the collection of CC5 MRSA from Rio de Janeiro studied here are presented in Table 3.

**Table 3:** Sensitivity, specificity, positive predictive value (PPV), and negative predictive value (NPV) for RdJ detection tests for the identification of ST105-SCC*mec*II (RdJ and non-RdJ) in the collection of *Staphylococcus aureus* studied.

Genotype	Detection of ST105-SCC*mec*II		Total
	Negative	Positive	
ST105-RdJ	0	78 (true)	78
ST105-non RdJ	4 (false)	0	4
ST4876	0	2 (false)	2
Other STs	128 (true)	0	128
Total	132	80	212
Specificity:	98.46% (94.55–98.81%)	PPV:	97.50% (90.78–93.65%)
Sensitivity:	95.12% (87.98–98.66%)	NPV:	96.97% (92.48–98.81%)

MLST and clone type (RdJ or non-RdJ) were performed using genomics and phylogenomics analyses. The parentheses correspond to the 95.00% confidence interval.

## 4 Discussion

We used a strategy of producing a binary (1,0) pangenome matrix based on the presence/absence of exact match ORFs to identify nucleotide apomorphies for rapid and simple tests for the detection of isolates belonging to the RdJ clone of MRSA. We demonstrated that the use of pangenomes is particularly powerful for identifying highly specific diagnostic tests for clonal identification. The tests developed here showed 100% specificity and sensitivity for the detection of MRSA isolates belonging to RdJ clone. Furthermore, the RdJ detection tests were equally accurate in identifying clonal complex 5 (CC5) isolates, the main clonal complex spread in Rio de Janeiro, which is also widespread in other countries including USA (Viana et al., 2021). Thus, because these tests are relatively simple and inexpensive compared with WGS, the RdJ detection tests can be used, for example, on a large scale to investigate the prevalence and incidence of the RdJ clone in hospital BSI, allowing comprehensive studies on the risks for its acquisition, and for morbidity and mortality associated with BSI caused by RdJ isolates. It is also notable that ST105-SCC*mec*II isolates increasingly have been reported more recently in hospitals in other Brazilian states and in other countries (Dabul and Camargo, 2014; Bes et al., 2021). Due to its prevalence in BSI in our region, the use of large-scale molecular tests to screen for RdJ is of great interest.

A search of the 71,017 annotated genomes deposited in NCBI GenBank database revealed that the vast majority of A1106G mutation in the *aur* gene (96.25%; *n* = 154/160) was in ST105 genomes or its variants. Although this specific mutation in non-CC5 genomes is rare, we have included specific primers in the RdJ detection tests to identify CC5. Thus, the presence of A1106G mutation in non-CC5 isolates will be revealed by the tests developed here, avoiding confusion in the identification of the RdJ clone, since the latter belongs to CC5. None of 9,555 ST5(CC5) genomes (close relatives of RdJ strains) carry A1106G mutation.

To find a biomarker to detect the RdJ clone, we initially established an arbitrary cutoff corresponding to a *p*-value < 10^−50^. However, if none of the 56 ORFs selected by this cutoff served as a good biomarker for designing a relatively simple molecular test, a higher cutoff could be attempted, but this could compromise the specificity of the biomarker. In this case, an alternative could be to use more than one biomarker to guarantee the specificity of the detection test. Nevertheless, the need to choose a not very specific biomarker seems unlikely, given the relatively high number of commercially available restriction endonucleases. For example, in the case of the RdJ detection tests, the ORF that presented the lowest *p* value (*aur*) proved to be ideal for detecting the RdJ clone.

Although most isolates from Rio de Janeiro and São Paulo belong to the RdJ clone, which is part of the ST105-SCC*me*cII lineage, this method was not designed and cannot be used for identification of other isolates of ST105-SCC*mec*II lineage that do not belong to the RdJ clone. For example, out of a total 1,385 ST105 in the collection of 71,017 *S. aureus* genomes, only 150 (10.83%) had the *aur* mutation. In addition, four ST105 variants [ST4876 (n = 2) and ST5288 (n = 2)] also clustered in RdJ clade. In fact, most ST105-SCC*mec*II isolates of international origin do not contain the *aur* (A1106G) mutation. Of the total of 71,017 *S. aureus* genomes analyzed, 5 were from Europe (Denmark, *n* = 3; Netherlands, *n* = 2) and 2 USA (city of Boston, *n* = 1 and New York City, *n* = 1).

In this context, research associating phylogenomics, genomic epidemiology and pangenomics tools is of paramount importance, not only to accelerate the understanding of evolutionary events and epidemiological factors that may be involved in the spread of hypervirulent and multidrug resistant pathogens, but also to provide simpler and economical tools (Uhlemann et al., 2014; Planet, 2017; Mirande et al., 2018). Such rapid tests can also be fundamental for designing protocols for more personalized and efficient treatment of patients, contributing to patient health safety (Rajapaksha et al., 2019).

Pickens et al. (2021) reported a dramatic decrease in the use of vancomycin as the drug of choice for the treatment of pneumonia in hospitals after the implementation of a simple PCR-based screening method to exclude the presence of MRSA. Considering that specific clones of MRSA, not infrequently, present different profiles of antimicrobial resistance, and that they may be involved in different epidemiologic and clinical scenarios and presentations (Côrtes et al., 2021; Viana et al., 2021), the use of rapid tests to identify hypervirulent, highly transmissible, well-adapted, or MDR clones is highly desirable.

Indeed, rapid molecular tests have been developed for the detection of MRSA lineages. A duplex PCR-based strategy was used to detect the livestock-associated MRSA (LA-MRSA) CC398 MRSA (Stegger et al., 2011). Also, a rapid test for the detection of the USA300 MRSA was developed resulting in a 100% match with the WGS data (Bonnstetter et al., 2007) This clone is an important, disseminated MRSA in hospital and community settings in the US (Planet, 2017). Other PCR-based tests have also been developed to identify MLST clonal complexes (Lindsay and Sung, 2010). The difference between the tests developed here and others is that the RdJ detection tests identify the clone, according to its phylogeny, while the others identify at most the MLST clonal complex.

In addition to the importance of RdJ detection tests for large-scale clinical and epidemiological investigations, we believe that scientists guided by the strategy used in this work will be able to easily perform analysis of pangenome ORFs to reveal apomorphies that can be used for the development of accurate diagnostic tests, not only for MRSA clones, but also for other pathogens, aiming at more effective treatment and successful control of infectious diseases.

## Data availability statement

The datasets presented in this study can be found in online repositories. The names of the repository/repositories and accession number(s) can be found in the article/supplementary material.

## Author contributions

ME: Methodology, Validation, Data curation, Formal Analysis, Investigation, Writing – original draft. AVi: Data curation, Formal Analysis, Investigation, Methodology, Writing – original draft. GV: Formal Analysis, Investigation, Writing – original draft. AB: Formal Analysis, Investigation, Writing – original draft. AM: Formal Analysis, Investigation, Writing – original draft. FM: Formal Analysis, Investigation, Writing – original draft. AFe: Formal Analysis, Investigation, Writing – original draft. AVe: Formal Analysis, Investigation, Writing – original draft. BF: Data curation, Project administration, Supervision, Writing – review & editing. PP: Data curation, Writing – review & editing, Formal Analysis, Funding acquisition, Investigation. AFi: Funding acquisition, Writing – review & editing, Conceptualization, Methodology, Project administration, Supervision, Validation.

## Footnotes

^1^ https://blast.ncbi.nlm.nih.gov

^2^ https://www.uniprot.org

^3^ http://www.microsoft.com

^4^ https://www.genomicepidemiology.org/services/

^5^ https://github.com/tseemann/mlst

^6^ https://realphy.unibas.ch/realphy/

^7^ https://www.geneious.com/features/phylogenetic-tree-building/

^8^ https://itol.embl.de/

^9^ http://araw.mede.uic.edu/cgi-bin/testcalc.pl
