# Supplementary material for: RdJ detection tests to identify a unique MRSA clone of ST105-SCCmecII lineage and its variants disseminated in the metropolitan region of Rio de Janeiro
Source: Front Microbiol. 2023 Nov 20;14:1275918. doi: 10.3389/fmicb.2023.1275918 (PMC10694290; doi:10.3389/fmicb.2023.1275918)
Supplement: Supplementary file 2 [file Table_2.DOCX]

**Supplementary Table S2**. List of open reading frame (ORF) obtained from the binary matrix analysis for the two groups of CC5 genomes (RdJ vs. non-RdJ). The cut-off value (*) was defined as -log10 *p*-value >50. The genome sequencing of the MRSA N315 strain was used as a reference.

| **ORF identification** | **log2 fold change** | **-log10 p valor** | **Above cut-off** |
| --- | --- | --- | --- |
| **Aur** | 1 | 270.3 | * |
| **Trpg** | 1 | 252.7 | * |
| **Pcka** | 1 | 245.3 | * |
| **Sa0849** | 0.9 | 226.9 | * |
| **Sa1437** | 1 | 226.9 | * |
| **Sa0667** | 1 | 212.3 | * |
| **Sa0828** | 0.9 | 146.4 | * |
| **Sa1280** | 0.9 | 146.1 | * |
| **Sa1687** | 0.9 | 146 | * |
| **Sa1590** | 0.7 | 144.6 | * |
| **Sa0706** | 0.9 | 143.6 | * |
| **Sa1125** | 0.9 | 139.1 | * |
| **Hemc** | 1 | 139.1 | * |
| **Sa1129** | 0.9 | 138.8 | * |
| **Sa0721** | 0.9 | 137.8 | * |
| **Sa2194** | 0.9 | 134.6 | * |
| **Fmta** | 0.9 | 132.5 | * |
| **Pros** | 0.9 | 132.4 | * |
| **Sa2100** | 0.9 | 129.6 | * |
| **Sa2437** | 0.9 | 129.6 | * |
| **Adhe** | 0.9 | 127 | * |
| **Sa0906** | 1 | 121.5 | * |
| **Sa2137** | 1 | 117.4 | * |
| **Prfc** | 0.9 | 117.3 | * |
| **Pdha** | 0.9 | 115.1 | * |
| **Sa1252** | 0.8 | 111.8 | * |
| **Crtm** | 0.9 | 108.8 | * |
| **Sa0936** | 0.9 | 107.9 | * |
| **Sa2347** | 0.9 | 106.8 | * |
| **Sa1168** | 0.9 | 106.8 | * |
| **Sa0817** | 0.9 | 103.9 | * |
| **Sa0019** | 0.9 | 103.9 | * |
| **Sa0019** | 0.9 | 103.9 | * |
| **Sa1054** | 0.9 | 100.3 | * |
| **Sa1693** | 0.9 | 100.2 | * |
| **Sa0904** | 1 | 97.1 | * |
| **Sa1619** | 0.8 | 96.8 | * |
| **Sa2379** | 0.9 | 96 | * |
| **Sa0233** | 0.7 | 95.1 | * |
| **Secf** | 0.8 | 94.3 | * |
| **Sa0342** | 0.9 | 93.5 | * |
| **Sa0673** | 0.8 | 92.7 | * |
| **Glca** | 0.7 | 86.6 | * |
| **Sa2453** | 0.9 | 86.6 | * |
| **Sa1838** | 0.8 | 85.9 | * |
| **Sa1655** | 0.6 | 83.7 | * |
| **Rpoc** | 0.8 | 83.7 | * |
| **Sa2195** | 0.8 | 81.3 | * |
| **Leus** | 0.8 | 79.6 | * |
| **Truncated(Mapw)** | 0.8 | 78.5 | * |
| **Sa2434** | 0.8 | 67.5 | * |
| **Sa2411** | 0.7 | 66.9 | * |
| **Sa1273** | 0.8 | 63.7 | * |
| **Lyth** | 0.7 | 60.1 | * |
| **Sa0026** | 0.5 | 50.4 | * |
| **Sa0034** | 0.5 | 50.4 | * |
| **Sa0282** | 0.7 | 45 |  |
| **Sa0749** | 0.7 | 38.6 |  |
| **Sa0677** | 0.7 | 38.2 |  |
| **Sa1534** | 0.7 | 37.9 |  |
| **Sa2285** | -1.2 | 37.7 |  |
| **Sa2365** | 0.7 | 37.5 |  |
| **Sa0104** | 0.7 | 37.5 |  |
| **Tyra** | 0.7 | 37.5 |  |
| **Sa2483** | 0.7 | 37.5 |  |
| **Sa1893** | 0.7 | 37.1 |  |
| **Sa1051** | 0.7 | 36.8 |  |
| **Sa1749** | 0.7 | 36.8 |  |
| **Sa2355** | 0.7 | 36.8 |  |
| **Aroa** | 0.7 | 36.4 |  |
| **Sa2020** | 0.7 | 36.4 |  |
| **Feob** | 0.7 | 36.4 |  |
| **Brab** | 0.7 | 36 |  |
| **Fhud2** | 0.7 | 35.7 |  |
| **Sa1354** | 0.7 | 35.7 |  |
| **Sa1932** | 0.7 | 34.3 |  |
| **Sa0754** | 0.7 | 33.9 |  |
| **Sa1431** | 0.7 | 33.2 |  |
| **Bfmbab** | 0.7 | 33.2 |  |
| **Sa1511** | 0.7 | 33.2 |  |
| **Clpb** | 0.7 | 33.2 |  |
| **Sa1216** | 0.6 | 33.2 |  |
| **Rpsb** | 0.7 | 32.9 |  |
| **Trap** | 0.7 | 31.9 |  |
| **Truncated-Tnp** | 0.4 | 31.5 |  |
| **Xprt** | 0.7 | 31.5 |  |
| **Vrag** | 0.7 | 31.2 |  |
| **Sa1788** | 0.6 | 31.2 |  |
| **Sa1785** | 0.4 | 30.2 |  |
| **Sa2415** | 0.7 | 29.2 |  |
| **Sa1320** | 0.4 | 28.9 |  |
| **Sa0428** | 0.7 | 28.9 |  |
| **Mete** | 0.6 | 28.6 |  |
| **Rocd** | 0.7 | 28.6 |  |
| **Sa1841** | 0.7 | 28.6 |  |
| **Rsbu** | 0.7 | 28.6 |  |
| **Sas090** | 0.5 | 28.3 |  |
| **Sa0089** | 0.6 | 27.6 |  |
| **Trpf** | 0.5 | 27 |  |
| **Nora** | 0.7 | 25.7 |  |
| **Spld** | 0.5 | 25.7 |  |
| **Sa0182** | 0.7 | 25.4 |  |
| **Sa0287** | 0.6 | 24.3 |  |
| **Sa1672** | 0.5 | 23 |  |
| **Leub** | 0.6 | 22.7 |  |
| **Splf** | 0.4 | 22.7 |  |
| **Set/08** | 0.5 | 22.4 |  |
| **Sa0959** | 0.6 | 22.4 |  |
| **Sas064** | 0.5 | 22.1 |  |
| **Sa2102** | 0.4 | 22.1 |  |
| **Dnag** | 0.5 | 21.8 |  |
| **Sa1793** | 0.4 | 21.6 |  |
| **Sa1243** | 0.5 | 21.5 |  |
| **Sa0043** | 0.2 | 20.1 |  |
| **Narg** | 0.4 | 19.8 |  |
| **Sa0303** | 0.3 | 19.8 |  |
| **Sa1664** | 0.7 | 19.4 |  |
| **Sa1773** | 0.6 | 18.7 |  |
| **Sa0645** | 0.6 | 18 |  |
| **Sa2404** | 0.7 | 17.2 |  |
| **Sa0720** | -0.7 | 17.1 |  |
| **Sa1772** | 0.3 | 17 |  |
| **Sa1771** | 0.3 | 16.7 |  |
| **Sa1778** | 0.3 | 16.7 |  |
| **Sa1762** | 0.3 | 16.4 |  |
| **Sa0083** | 0.5 | 16.4 |  |
| **Sas060** | 0.3 | 15.9 |  |
| **Sas061** | 0.3 | 15.9 |  |
| **Sa1071** | 0.6 | 15.8 |  |
| **Sa1763** | 0.2 | 15.6 |  |
| **Sa1806** | 0.3 | 15.6 |  |
| **Sep** | 0.2 | 15.4 |  |
| **Sak** | 0.2 | 15.1 |  |
| **Sa2317** | 0.6 | 14.8 |  |
| **Sa1777** | 0.3 | 14.8 |  |
| **Sa1803** | 0.3 | 14.6 |  |
| **Sa1768** | 0.2 | 14.6 |  |
| **Sa1808** | 0.2 | 14.6 |  |
| **Sa1802** | 0.2 | 14.3 |  |
| **Ffh** | 0.6 | 14.2 |  |
| **Tagh** | 0.6 | 14.1 |  |
| **Sa1805** | 0.2 | 14 |  |
| **Sa1759** | 0.2 | 13.8 |  |
| **Sa1770** | 0.3 | 13.8 |  |
| **Sa1774** | 0.3 | 13.8 |  |
| **Sa1809** | 0.2 | 13.8 |  |
| **Int** | 0.2 | 13.8 |  |
| **Sa1661** | 0.3 | 13.2 |  |
| **Sa1760** | 0.3 | 12.9 |  |
| **Sa0078** | 0.4 | 12.8 |  |
| **Sa1807** | 0.2 | 12.7 |  |
| **Sa0967** | -0.2 | 12.2 |  |
| **Sa0836** | 0.5 | 11.8 |  |
| **Trpa** | -0.2 | 11.7 |  |
| **Sa2357** | -0.6 | 11.7 |  |
| **Menc** | -0.4 | 11.5 |  |
| **Sa1801** | 0.2 | 11.4 |  |
| **Sa0969** | 0.6 | 11.3 |  |
| **Sa0299** | 0.5 | 11.2 |  |
| **Sa1775** | 0.3 | 11.1 |  |
| **Sas036** | 0.2 | 10.9 |  |
| **Coa** | 0.2 | 10.8 |  |
| **Sa1105** | 0.5 | 10.3 |  |
| **Sa0930** | 0.2 | 10.1 |  |
| **Truncated(Lyta)** | 0.2 | 10.1 |  |
| **Sa1670** | 0.5 | 9.9 |  |
| **Sa1766** | 0.1 | 9.9 |  |
| **Sa1755** | 0.2 | 9.9 |  |
| **Sa0834** | 0.4 | 9.8 |  |
| **Sa1786** | 0.3 | 9.7 |  |
| **Sa1776** | 0.2 | 9.6 |  |
| **Sa0774** | 0.5 | 9.6 |  |
| **Sa0275** | -0.4 | 9.6 |  |
| **Moaa** | -0.2 | 9.5 |  |
| **Sa1714** | 0.4 | 9.2 |  |
| **Copa** | -0.6 | 9 |  |
| **Pbp3** | 0.4 | 8.9 |  |
| **Recn** | 0.5 | 8.9 |  |
| **Dapb** | 0.6 | 8.9 |  |
| **Sa1764** | 0.2 | 8.9 |  |
| **Gntp** | 0.5 | 8.9 |  |
| **Sa1001** | 0.4 | 8.8 |  |
| **Sa0700** | 0.4 | 8.8 |  |
| **Sa2311** | -0.3 | 8.8 |  |
| **Sspa** | -0.4 | 8.8 |  |
| **Sa1618** | -0.5 | 8.7 |  |
| **Sa2265** | 0.4 | 8.7 |  |
| **Sa1389** | 0.6 | 8.6 |  |
| **Sa2394** | 0.6 | 8.6 |  |
| **Acsa** | 0.6 | 8.4 |  |
| **Fmtb(Mrp)** | 0.4 | 8.4 |  |
| **Sa0172** | 0.6 | 8.4 |  |
| **Argc** | 0.6 | 8.4 |  |
| **Sa0223** | 0.5 | 8.4 |  |
| **Sa0417** | 0.6 | 8.4 |  |
| **Mets** | 0.5 | 8.4 |  |
| **Sa0463** | 0.6 | 8.4 |  |
| **Sa0613** | 0.5 | 8.4 |  |
| **Cdr** | 0.5 | 8.4 |  |
| **Sa0833** | 0.6 | 8.4 |  |
| **Sa0866** | 0.6 | 8.4 |  |
| **Sa0916** | 0.5 | 8.4 |  |
| **Cfxe** | 0.6 | 8.4 |  |
| **Sas039** | 0.6 | 8.4 |  |
| **Thrc** | 0.5 | 8.4 |  |
| **Sa1209** | 0.6 | 8.4 |  |
| **Lysc** | 0.5 | 8.4 |  |
| **Gnd** | 0.6 | 8.4 |  |
| **Sa1401** | 0.6 | 8.4 |  |
| **Gapb** | 0.6 | 8.4 |  |
| **Sa1679** | 0.5 | 8.4 |  |
| **Sa1680** | 0.5 | 8.4 |  |
| **Sa1686** | 0.6 | 8.4 |  |
| **Sgtb** | 0.6 | 8.4 |  |
| **Aldh** | 0.6 | 8.4 |  |
| **Kdpa** | 0.5 | 8.4 |  |
| **Kdpe** | 0.6 | 8.4 |  |
| **Sa1957** | 0.6 | 8.4 |  |
| **Urec** | 0.5 | 8.4 |  |
| **Phob** | 0.6 | 8.4 |  |
| **Icab** | 0.6 | 8.4 |  |
| **Sa2474** | 0.5 | 8.4 |  |
| **Lpl3** | 0.4 | 8.3 |  |
| **Sa2180** | 0.6 | 8.2 |  |
| **Capi** | 0.5 | 8.2 |  |
| **Sa0890** | 0.6 | 8.2 |  |
| **Sa1983** | 0.5 | 8.2 |  |
| **Sa2339** | 0.5 | 8.2 |  |
| **Pare** | -0.3 | 8.1 |  |
| **Sa1754** | 0.3 | 8.1 |  |
| **Isdg** | 0.6 | 7.9 |  |
| **Sa1990** | 0.6 | 7.9 |  |
| **Sa1733** | 0.4 | 7.9 |  |
| **Nth** | -0.7 | 7.8 |  |
| **Scda** | -0.5 | 7.7 |  |
| **Sa1157** | 0.4 | 7.7 |  |
| **Sa2279** | 0.5 | 7.7 |  |
| **Sarh1** | 0.4 | 7.6 |  |
| **Glck** | -0.8 | 7.6 |  |
| **Mtlf** | -0.6 | 7.6 |  |
| **Sa1919** | 0.3 | 7.5 |  |
| **Truncated-SA** | 0.4 | 7.5 |  |
| **Sa2266** | 0.4 | 7.4 |  |
| **Sa1795** | 0.2 | 7.3 |  |
| **Hsdr** | -0.7 | 7.3 |  |
| **Set/11** | 0.4 | 7.2 |  |
| **Sa0241** | 0.4 | 7.2 |  |
| **Sa1654** | 0.4 | 7.2 |  |
| **Accc** | -0.3 | 7.2 |  |
| **Tnp** | 0.3 | 7.2 |  |
| **Kdpb** | -0.2 | 7 |  |
| **Sa1753** | 0.2 | 6.8 |  |
| **Sa1334** | -0.2 | 6.8 |  |
| **Sa0298** | -0.5 | 6.8 |  |
| **Sa2278** | -0.5 | 6.8 |  |
| **Sa1783** | -0.8 | 6.8 |  |
| **Sa0308** | -0.5 | 6.7 |  |
| **Sa1961** | 0.3 | 6.7 |  |
| **Sa1796** | 0.3 | 6.7 |  |
| **Arca** | 0.4 | 6.6 |  |
| **Seca** | 0.3 | 6.6 |  |
| **Sa0306** | -0.5 | 6.6 |  |
| **Vrab** | -0.4 | 6.6 |  |
| **Lukd** | -0.2 | 6.6 |  |
| **Sa0333** | -0.5 | 6.6 |  |
| **Sas018** | 0.4 | 6.6 |  |
| **Pbp4** | 0.3 | 6.5 |  |
| **Sdrd** | -0.3 | 6.5 |  |
| **Geh** | -0.3 | 6.4 |  |
| **Set/09** | -0.6 | 6.4 |  |
| **Trpb** | 0.3 | 6.3 |  |
| **Map** | -0.1 | 6.2 |  |
| **Sa1374** | -0.2 | 6.2 |  |
| **Sa0854** | -0.5 | 6.2 |  |
| **Sa1379** | 0.4 | 6.2 |  |
| **Sa0779** | 0.3 | 6.1 |  |
| **Purf** | -0.5 | 6.1 |  |
| **Sa0975** | 0.4 | 6.1 |  |
| **Sa1085** | -0.2 | 5.9 |  |
| **Frua** | -0.5 | 5.9 |  |
| **Sa0276** | -0.6 | 5.8 |  |
| **Sa0185** | -0.3 | 5.7 |  |
| **Orfx** | -0.6 | 5.7 |  |
| **Citc** | 0.3 | 5.7 |  |
| **Sa1459** | -0.2 | 5.7 |  |
| **Sa1592** | -0.4 | 5.7 |  |
| **Sa1451** | -0.1 | 5.7 |  |
| **Atl** | -0.5 | 5.6 |  |
| **Sa1580** | -0.5 | 5.6 |  |
| **Sa1584** | -0.1 | 5.5 |  |
| **Sa2402** | -0.6 | 5.4 |  |
| **Sa0289** | 0.3 | 5.4 |  |
| **Sa0640** | 0.3 | 5.4 |  |
| **Splc** | -0.6 | 5.4 |  |
| **Sa0406** | -0.6 | 5.4 |  |
| **Dnax** | -0.2 | 5.4 |  |
| **Sa0349** | -0.4 | 5.3 |  |
| **Asps** | -0.1 | 5.3 |  |
| **Acua** | -0.4 | 5.3 |  |
| **Sa2368** | -0.2 | 5.3 |  |
| **Appf** | -0.5 | 5.3 |  |
| **Sa0065** | -1 | 5.2 |  |
| **Sa0097** | -0.5 | 5.2 |  |
| **Sa1564** | 0.4 | 5.2 |  |
| **Sa1257** | -0.4 | 5.2 |  |
| **Sa0790** | -0.4 | 5.2 |  |
| **Glvc** | -0.5 | 5.2 |  |
| **Sa1765** | -0.9 | 5.2 |  |
| **Sa1562** | -0.7 | 5.2 |  |
| **Pdhb** | -0.4 | 5.1 |  |
| **Sa2164** | -0.6 | 5.1 |  |
| **Iles** | -0.2 | 5.1 |  |
| **Kdpc(Sccmec)** | -1 | 5 |  |
| **Sa1321** | 0.3 | 5 |  |
| **Sa2120** | -0.1 | 4.9 |  |
| **Srta** | -0.1 | 4.9 |  |
| **Sa1276** | -0.4 | 4.9 |  |
| **Sa1386** | -0.5 | 4.9 |  |
| **Rlub** | -0.1 | 4.9 |  |
| **Sa1702** | -0.4 | 4.8 |  |
| **Huth** | -0.1 | 4.8 |  |
| **Sa1767** | -0.7 | 4.8 |  |
| **Sa0412** | -0.2 | 4.8 |  |
| **Sa1779** | 0 | 4.7 |  |
| **Sa1581** | -0.4 | 4.7 |  |
| **Sa0955** | -0.1 | 4.7 |  |
| **Sa0042** | -1 | 4.7 |  |
| **Thdf** | -0.4 | 4.7 |  |
| **Sa1269** | -0.1 | 4.7 |  |
| **Sa0044** | -1 | 4.7 |  |
| **Sa1398** | -0.4 | 4.7 |  |
| **Sa0094** | 0.3 | 4.7 |  |
| **Sa0783** | -0.5 | 4.6 |  |
| **Sa0271** | 0.3 | 4.6 |  |
| **Glmm(Femd)** | 0.3 | 4.6 |  |
| **Sas005** | 0.3 | 4.5 |  |
| **Sa1436** | -0.3 | 4.5 |  |
| **Sa0586** | 0.2 | 4.5 |  |
| **Sa0787** | 0.2 | 4.5 |  |
| **Sa1006** | 0.2 | 4.5 |  |
| **Sa1798** | -0.8 | 4.5 |  |
| **Crtn** | -0.7 | 4.4 |  |
| **Sa0332** | -0.1 | 4.4 |  |
| **Phes** | -0.4 | 4.4 |  |
| **Sa1572** | -0.4 | 4.4 |  |
| **Sa1575** | -0.3 | 4.4 |  |
| **Sa1034** | 0.2 | 4.4 |  |
| **Sa0913** | 0.2 | 4.3 |  |
| **Sa1804** | 0.1 | 4.3 |  |
| **Smc** | 0.3 | 4.3 |  |
| **Sa0623** | -0.1 | 4.2 |  |
| **Sa0688** | -0.4 | 4.2 |  |
| **Sa2217** | 0.3 | 4.2 |  |
| **Scra** | -0.5 | 4.2 |  |
| **Sa1924** | -0.4 | 4.2 |  |
| **Sa1050** | -0.1 | 4.2 |  |
| **Buta** | -0.1 | 4.2 |  |
| **Sa2158** | -0.1 | 4.2 |  |
| **Hemd** | -0.1 | 4.2 |  |
| **Kdpe(Sccmec)** | -1 | 4.2 |  |
| **Capl** | -0.5 | 4.2 |  |
| **Sa2367** | -0.4 | 4.2 |  |
| **Dra** | 0.2 | 4.1 |  |
| **Clfa** | 0.1 | 4 |  |
| **Clpx** | -0.2 | 4 |  |
| **Sa0987** | -0.5 | 4 |  |
| **Sa1969** | -0.2 | 4 |  |
| **Asns** | -0.4 | 4 |  |
| **Sa1143** | -0.4 | 4 |  |
| **Gntk** | -0.2 | 4 |  |
| **Sa0168** | -0.4 | 4 |  |
| **Atpa** | -0.2 | 4 |  |
| **Rpob** | -0.2 | 3.9 |  |
| **Rlp** | -0.2 | 3.9 |  |
| **Sa1967** | -0.1 | 3.9 |  |
| **Sa0180** | -0.4 | 3.9 |  |
| **Sa0605** | -0.4 | 3.9 |  |
| **Sa1344** | -0.4 | 3.9 |  |
| **Pyrp** | -0.5 | 3.9 |  |
| **Sa1784** | 0.2 | 3.9 |  |
| **Fabi** | -0.5 | 3.8 |  |
| **Oppf** | -0.1 | 3.8 |  |
| **Alas** | 0.2 | 3.8 |  |
| **Sa0367** | -0.5 | 3.7 |  |
| **Arob** | -0.4 | 3.7 |  |
| **Sa1970** | -0.5 | 3.7 |  |
| **Folp** | -0.4 | 3.7 |  |
| **Sa0850** | -0.3 | 3.7 |  |
| **Sas003** | -0.9 | 3.7 |  |
| **Sa0420** | -0.7 | 3.7 |  |
| **Sa1118** | -0.1 | 3.7 |  |
| **Capp** | -0.1 | 3.7 |  |
| **Mnhc** | 0.2 | 3.6 |  |
| **Sa1406** | -0.4 | 3.6 |  |
| **Sa0331** | -0.4 | 3.5 |  |
| **Sa1037** | -0.4 | 3.5 |  |
| **Fabg** | -0.1 | 3.5 |  |
| **Dapa** | -0.1 | 3.5 |  |
| **Sa1574** | -0.1 | 3.5 |  |
| **Sas080** | 0.2 | 3.5 |  |
| **Sa2142** | -0.4 | 3.5 |  |
| **Sa0334** | -0.2 | 3.4 |  |
| **Polc** | -0.2 | 3.4 |  |
| **Arcb** | -0.4 | 3.4 |  |
| **Sa0773** | -0.1 | 3.4 |  |
| **Acka** | -0.3 | 3.4 |  |
| **Mnha** | 0.3 | 3.4 |  |
| **Sa0226** | -0.5 | 3.3 |  |
| **Phor** | -0.1 | 3.3 |  |
| **Sa0857** | -0.1 | 3.3 |  |
| **Sa1611** | -0.1 | 3.3 |  |
| **Rpse** | -0.4 | 3.3 |  |
| **Sa2104** | -0.5 | 3.3 |  |
| **Sa2161** | -0.4 | 3.3 |  |
| **Sa2058** | -0.1 | 3.3 |  |
| **Sa2261** | -0.4 | 3.3 |  |
| **Sa0421** | -0.1 | 3.3 |  |
| **Sa1683** | -0.6 | 3.3 |  |
| **Sa1092** | 0.5 | 3.3 |  |
| **Purl** | -0.1 | 3.3 |  |
| **Sa1601** | -0.1 | 3.3 |  |
| **Sa0045** | -0.9 | 3.3 |  |
| **Sa1147** | -0.2 | 3.3 |  |
| **Sa1474** | -0.5 | 3.3 |  |
| **Opucb** | 0.2 | 3.2 |  |
| **Sa0933** | 0.2 | 3.2 |  |
| **Sa0046** | -0.9 | 3.2 |  |
| **Gatc** | -0.4 | 3.2 |  |
| **Dnaa** | -0.4 | 3.2 |  |
| **Urea** | -0.4 | 3.2 |  |
| **Sa0056** | -0.3 | 3.1 |  |
| **Sa2220** | -0.2 | 3.1 |  |
| **Sa1133** | -0.4 | 3.1 |  |
| **Sa0585** | -0.1 | 3.1 |  |
| **Sa0093** | -0.4 | 3.1 |  |
| **Sa0784** | 0.1 | 3.1 |  |
| **Thim** | 0.1 | 3.1 |  |
| **Sa0290** | 0.3 | 3.1 |  |
| **Ding** | -0.5 | 3.1 |  |
| **Sa0102** | -0.5 | 3.1 |  |
| **Sa0077** | -0.9 | 3 |  |
| **Dnab** | -0.1 | 3 |  |
| **Sa2371** | -0.2 | 3 |  |
| **Sas081** | -0.4 | 3 |  |
| **Sa2055** | -0.4 | 3 |  |
| **Ahpc** | -0.1 | 3 |  |
| **Sa1730** | -0.4 | 3 |  |
| **Rpsj** | -0.1 | 3 |  |
| **Argh** | -0.4 | 3 |  |
| **Sa1719** | -0.4 | 3 |  |
| **Rnr** | -0.6 | 2.9 |  |
| **Cysk** | -0.4 | 2.9 |  |
| **Sa0648** | -0.1 | 2.9 |  |
| **Sa0263** | -0.4 | 2.9 |  |
| **Sa0600** | 0.2 | 2.9 |  |
| **Cmk** | -0.1 | 2.8 |  |
| **Aapa** | -0.1 | 2.8 |  |
| **Sa2143** | -0.2 | 2.8 |  |
| **Sa2130** | -0.1 | 2.8 |  |
| **Pgi** | -0.2 | 2.8 |  |
| **Ampa** | -0.1 | 2.8 |  |
| **Truncated(Tnp)** | 0.2 | 2.8 |  |
| **Lpl6** | -0.1 | 2.8 |  |
| **Trub** | 0 | 2.8 |  |
| **Sa0054** | -0.9 | 2.8 |  |
| **Sa0076** | -0.9 | 2.8 |  |
| **Sa1061** | -0.4 | 2.8 |  |
| **Glya** | -0.4 | 2.8 |  |
| **Sa0272** | -0.5 | 2.8 |  |
| **Hsds** | -0.5 | 2.8 |  |
| **Sa1134** | -0.1 | 2.7 |  |
| **Fhs** | -0.4 | 2.7 |  |
| **Tcab** | -0.4 | 2.7 |  |
| **Rply** | -0.1 | 2.7 |  |
| **Sa0074** | -0.9 | 2.7 |  |
| **Srra** | -0.4 | 2.7 |  |
| **Hu** | -0.4 | 2.7 |  |
| **Sa1617** | -0.4 | 2.7 |  |
| **Sa0394** | -0.1 | 2.7 |  |
| **Modb** | -0.1 | 2.7 |  |
| **Sa2095** | -0.1 | 2.7 |  |
| **Sa1626** | 0.2 | 2.7 |  |
| **Sa0195** | 0.1 | 2.6 |  |
| **Kdpa(Sccmec)** | -0.9 | 2.6 |  |
| **Sa1167** | 0.2 | 2.6 |  |
| **Sa0075** | -0.9 | 2.6 |  |
| **Sa1563** | -0.1 | 2.6 |  |
| **Hisf** | -0.2 | 2.6 |  |
| **Sa1440** | -0.2 | 2.6 |  |
| **Sa1178** | -0.1 | 2.6 |  |
| **Sas044** | 0.1 | 2.6 |  |
| **Sa0931** | -0.4 | 2.5 |  |
| **Sa0073** | -0.9 | 2.5 |  |
| **Vrae** | -0.5 | 2.5 |  |
| **Sa2153** | -0.1 | 2.5 |  |
| **Sa1135** | -0.1 | 2.5 |  |
| **Sa0578** | 0.1 | 2.5 |  |
| **Sa2011** | 0 | 2.5 |  |
| **Sa1853** | 0 | 2.5 |  |
| **Sa1737** | 0.3 | 2.4 |  |
| **Dnae** | -0.3 | 2.4 |  |
| **Sa0060** | -0.9 | 2.4 |  |
| **Sa1069** | -0.1 | 2.4 |  |
| **Sa2200** | -0.4 | 2.4 |  |
| **Sa0478** | -0.1 | 2.4 |  |
| **Sa0634** | -0.4 | 2.4 |  |
| **Sa0705** | -0.2 | 2.4 |  |
| **Opp-1D** | -0.2 | 2.4 |  |
| **Sa1009** | -0.4 | 2.4 |  |
| **Fbp** | -0.4 | 2.4 |  |
| **Sirc** | -0.4 | 2.4 |  |
| **Hld** | 0.1 | 2.4 |  |
| **Sa2175** | -0.1 | 2.4 |  |
| **Sa0838** | 0.1 | 2.4 |  |
| **Sa1708** | 0.1 | 2.4 |  |
| **Hemb** | -0.2 | 2.4 |  |
| **Opp-2B** | -0.6 | 2.3 |  |
| **Amps** | -0.4 | 2.3 |  |
| **Reca** | -0.1 | 2.3 |  |
| **Sa0318** | -0.1 | 2.3 |  |
| **Riba** | -0.4 | 2.3 |  |
| **Sa2436** | -0.2 | 2.3 |  |
| **Drm** | -0.1 | 2.3 |  |
| **Sa0680** | -0.1 | 2.3 |  |
| **Sa0887** | -0.4 | 2.3 |  |
| **Lacb** | -0.4 | 2.3 |  |
| **Sa1741** | 0.2 | 2.3 |  |
| **Sa0482** | -0.2 | 2.2 |  |
| **Sa2061** | -0.1 | 2.2 |  |
| **Xylr** | -0.9 | 2.2 |  |
| **Sa1641** | -0.4 | 2.2 |  |
| **Sa0891** | 0.1 | 2.2 |  |
| **Sa2263** | 0.2 | 2.2 |  |
| **Sa2457** | -0.5 | 2.2 |  |
| **Sa0072** | -0.9 | 2.2 |  |
| **Sa1840** | -0.1 | 2.2 |  |
| **Sa0055** | -0.9 | 2.2 |  |
| **Sa0717** | -0.4 | 2.2 |  |
| **Sa0187** | 0.1 | 2.2 |  |
| **Moae** | 0.1 | 2.2 |  |
| **Nrdd** | -0.1 | 2.2 |  |
| **Sa0865** | -0.1 | 2.2 |  |
| **Sa0658** | -0.4 | 2.2 |  |
| **Opp-1C** | -0.4 | 2.2 |  |
| **Sa2126** | -0.1 | 2.2 |  |
| **Sa1233** | 0.1 | 2.2 |  |
| **Sa1931** | 0.1 | 2.2 |  |
| **Sa1780** | -0.4 | 2.1 |  |
| **Sa0202** | -0.1 | 2.1 |  |
| **Infb** | -0.1 | 2.1 |  |
| **Sa0327** | -0.1 | 2.1 |  |
| **Rimm** | 0 | 2.1 |  |
| **Sa1573** | -0.1 | 2.1 |  |
| **Sa2221** | -0.4 | 2.1 |  |
| **Sa0064** | -0.9 | 2.1 |  |
| **Glna** | -0.1 | 2.1 |  |
| **Sa1633** | -0.2 | 2.1 |  |
| **Sa2080** | -0.1 | 2 |  |
| **Hprk** | -0.1 | 2 |  |
| **Lacg** | 0.2 | 2 |  |
| **Sa1726** | 0.1 | 2 |  |
| **Sas087** | 0.1 | 2 |  |
| **Kdpb(Sccmec)** | -0.9 | 2 |  |
| **Sa0839** | 0.1 | 2 |  |
| **Sa1688** | 0.1 | 2 |  |
| **Sa1789** | -0.6 | 2 |  |
| **Cape** | 0.1 | 2 |  |
| **Sa1978** | 0.1 | 2 |  |
| **Sa1196** | -0.1 | 2 |  |
| **Sa0262** | -0.4 | 2 |  |
| **Rada** | -0.4 | 2 |  |
| **Scrr** | -0.1 | 2 |  |
| **Sa1943** | -0.3 | 2 |  |
| **Sa1475** | -0.1 | 2 |  |
| **Sa2111** | -0.1 | 2 |  |
| **Pyca** | -0.1 | 1.9 |  |
| **Sa0568** | 0.1 | 1.9 |  |
| **Sa0582** | 0.1 | 1.9 |  |
| **Sa1986** | -0.4 | 1.9 |  |
| **Ured** | -0.1 | 1.9 |  |
| **Sa2099** | -0.2 | 1.9 |  |
| **Sa2117** | -0.4 | 1.9 |  |
| **Sa0270** | 0 | 1.9 |  |
| **Sa0554** | 0.1 | 1.9 |  |
| **Sa0556** | 0.1 | 1.9 |  |
| **Sa2107** | 0.1 | 1.9 |  |
| **Sa0035** | 0.3 | 1.9 |  |
| **Sa0209** | -0.4 | 1.9 |  |
| **Sa0293** | -0.4 | 1.9 |  |
| **Gltc** | -0.1 | 1.9 |  |
| **Sa2109** | -0.1 | 1.9 |  |
| **Sa1558** | -0.4 | 1.9 |  |
| **Alda** | 0.1 | 1.8 |  |
| **Uvrc** | -0.1 | 1.8 |  |
| **Sa2152** | -0.1 | 1.8 |  |
| **Gtab** | -0.1 | 1.8 |  |
| **Heml** | -0.1 | 1.8 |  |
| **Cdsa** | -0.1 | 1.8 |  |
| **Thya** | -0.1 | 1.8 |  |
| **Opud** | -0.1 | 1.8 |  |
| **Lysp** | -0.1 | 1.8 |  |
| **Moac** | -0.1 | 1.8 |  |
| **Yent2** | 0.2 | 1.8 |  |
| **Sa0379** | 0.1 | 1.7 |  |
| **Sira** | -0.4 | 1.7 |  |
| **Sa1314** | -0.1 | 1.7 |  |
| **Naga** | -0.4 | 1.7 |  |
| **Sa0096** | -0.1 | 1.7 |  |
| **Sa0860** | -0.1 | 1.7 |  |
| **Fmhc(Eprh)** | 0.1 | 1.7 |  |
| **Cyss** | -0.2 | 1.7 |  |
| **Sa2269** | 0.2 | 1.7 |  |
| **Sa0653** | 0.1 | 1.7 |  |
| **Sbnb** | -0.1 | 1.7 |  |
| **Dhom** | -0.1 | 1.7 |  |
| **Sas059** | -0.1 | 1.7 |  |
| **Sa2358** | -0.1 | 1.7 |  |
| **Sa0358** | -0.1 | 1.7 |  |
| **Sa0703** | -0.1 | 1.7 |  |
| **Sa1867** | -0.1 | 1.7 |  |
| **Fmha** | -0.1 | 1.7 |  |
| **Sa0851** | -0.1 | 1.7 |  |
| **Sa0310** | -0.1 | 1.7 |  |
| **Sa0571** | 0.1 | 1.7 |  |
| **Sa0286** | 0.1 | 1.7 |  |
| **Sa1877** | -0.1 | 1.6 |  |
| **Sa0264** | -0.4 | 1.6 |  |
| **Murz** | -0.1 | 1.6 |  |
| **Sa0663** | 0.1 | 1.6 |  |
| **Mend** | 0 | 1.6 |  |
| **Rpsn** | 0.1 | 1.6 |  |
| **Sa0691** | 0.1 | 1.6 |  |
| **Sa0268** | 0.1 | 1.6 |  |
| **Spla** | -0.4 | 1.6 |  |
| **Sa2497** | -0.1 | 1.6 |  |
| **Pura** | -0.1 | 1.6 |  |
| **Mnhg** | -0.1 | 1.6 |  |
| **Sa2380** | -0.1 | 1.6 |  |
| **Sa0224** | -0.1 | 1.6 |  |
| **Sa1429** | 0.1 | 1.6 |  |
| **Sa2314** | 0.1 | 1.6 |  |
| **Sa0346** | -0.1 | 1.5 |  |
| **Sa0179** | -0.1 | 1.5 |  |
| **Sa2271** | -0.1 | 1.5 |  |
| **Sa1319** | 0.2 | 1.5 |  |
| **Sa0246** | 0.1 | 1.5 |  |
| **Set/10** | -0.1 | 1.5 |  |
| **Sa1162** | -0.1 | 1.5 |  |
| **Sa1546** | -0.4 | 1.5 |  |
| **Sa1888** | -0.2 | 1.5 |  |
| **Fmhb** | -0.1 | 1.5 |  |
| **Sa0883** | 0.1 | 1.5 |  |
| **Sa0518** | 0 | 1.5 |  |
| **Vrac** | 0.1 | 1.5 |  |
| **Sa0696** | 0.1 | 1.5 |  |
| **Sem** | -0.3 | 1.5 |  |
| **Sa0196** | 0.1 | 1.5 |  |
| **Miaa** | -0.1 | 1.5 |  |
| **Sa1565** | -0.1 | 1.5 |  |
| **Sa0280** | -0.1 | 1.5 |  |
| **Sa2260** | -0.1 | 1.5 |  |
| **Sa2489** | -0.1 | 1.5 |  |
| **Sa0630** | 0.1 | 1.5 |  |
| **Icar** | 0.1 | 1.5 |  |
| **Sa0619** | 0.1 | 1.5 |  |
| **Sa1658** | 0.1 | 1.5 |  |
| **Opuca** | -0.4 | 1.4 |  |
| **Sa0321** | -0.1 | 1.4 |  |
| **Sa1526** | -0.1 | 1.4 |  |
| **Sa1154** | -0.1 | 1.4 |  |
| **Sa1434** | -0.1 | 1.4 |  |
| **Pgk** | -0.2 | 1.4 |  |
| **Clpc** | 0.1 | 1.4 |  |
| **Sa0184** | -0.1 | 1.4 |  |
| **Clpl** | -0.1 | 1.4 |  |
| **Sa2305** | -0.1 | 1.4 |  |
| **Sa1255** | 0.1 | 1.4 |  |
| **Sa0166** | 0.1 | 1.4 |  |
| **Sa0208** | 0.1 | 1.4 |  |
| **Sa1797** | -0.5 | 1.4 |  |
| **Sas048** | 0.1 | 1.4 |  |
| **Hlgc** | -0.3 | 1.4 |  |
| **Sa1181** | -0.1 | 1.4 |  |
| **Sa0590** | 0.1 | 1.4 |  |
| **Sa1918** | 0 | 1.4 |  |
| **Sa1524** | 0.1 | 1.4 |  |
| **Sa1428** | 0.1 | 1.4 |  |
| **Sa1781** | -0.4 | 1.4 |  |
| **Uvrb** | -0.1 | 1.4 |  |
| **Sa1180** | -0.1 | 1.4 |  |
| **Moea** | -0.1 | 1.3 |  |
| **Sbi** | -0.1 | 1.3 |  |
| **Topb** | -0.1 | 1.3 |  |
| **Htra** | -0.2 | 1.3 |  |
| **Sa1060** | -0.1 | 1.3 |  |
| **Sa2441** | -0.1 | 1.3 |  |
| **Hish** | 0 | 1.3 |  |
| **Sa0732** | -0.1 | 1.3 |  |
| **Ssp** | -0.1 | 1.3 |  |
| **Sa0707** | 0 | 1.3 |  |
| **Capc** | 0 | 1.3 |  |
| **Sa0737** | 0 | 1.3 |  |
| **Sa0772** | 0 | 1.3 |  |
| **Atpc** | 0 | 1.3 |  |
| **Sas072** | 0.1 | 1.3 |  |
| **Sa2138** | 0 | 1.3 |  |
| **Sa0768** | -0.2 | 1.3 |  |
| **Sa1769** | -0.3 | 1.3 |  |
| **Icaa** | 0.1 | 1.3 |  |
| **Sa0502** | 0.1 | 1.3 |  |
| **Sa1597** | 0.1 | 1.3 |  |
| **Sa1123** | 0 | 1.3 |  |
| **Trps** | -0.1 | 1.3 |  |
| **Purm** | -0.1 | 1.3 |  |
| **Sa0433** | -0.1 | 1.3 |  |
| **Sa2060** | -0.1 | 1.3 |  |
| **Sa0555** | 0.1 | 1.3 |  |
| **Sa0886** | 0 | 1.3 |  |
| **Sa1208** | 0.1 | 1.3 |  |
| **Vick** | -0.5 | 1.3 |  |
| **Sas092** | 0 | 1.3 |  |
| **Mnaa** | 0.1 | 1.3 |  |
| **Sa0345** | 0 | 1.3 |  |
| **Sa1746** | 0.1 | 1.3 |  |
| **Atpd** | 0.1 | 1.3 |  |
| **Nusg** | 0.1 | 1.3 |  |
| **Tufa** | 0 | 1.3 |  |
| **Sa1616** | 0.1 | 1.3 |  |
| **Sa1977** | 0.1 | 1.3 |  |
| **Sa2418** | 0.1 | 1.3 |  |
| **Sa2397** | -0.2 | 1.2 |  |
| **Splb** | -0.1 | 1.2 |  |
| **Sa2272** | -0.1 | 1.2 |  |
| **Argg** | -0.1 | 1.2 |  |
| **Kdpd** | -0.1 | 1.2 |  |
| **Sa0141** | -0.1 | 1.2 |  |
| **Sa2123** | 0 | 1.2 |  |
| **Sa1823** | 0 | 1.2 |  |
| **Tmk** | 0 | 1.2 |  |
| **Sas021** | 0 | 1.2 |  |
| **Sa1684** | 0.1 | 1.2 |  |
| **Sa1845** | 0 | 1.2 |  |
| **Sas070** | 0 | 1.2 |  |
| **Sa2096** | 0 | 1.2 |  |
| **Tcar** | 0 | 1.2 |  |
| **Sa2378** | 0 | 1.2 |  |
| **Sa1800** | -0.3 | 1.2 |  |
| **Sa0371** | 0.1 | 1.2 |  |
| **Sa1743** | 0.1 | 1.2 |  |
| **Sa0197** | 0 | 1.2 |  |
| **Sa0499** | 0 | 1.2 |  |
| **Sara** | 0.1 | 1.2 |  |
| **Sa1274** | -0.1 | 1.2 |  |
| **Sa1794** | -0.6 | 1.2 |  |
| **Fmtc** | -0.1 | 1.2 |  |
| **Sa0701** | -0.1 | 1.2 |  |
| **Sa0745** | -0.4 | 1.2 |  |
| **Sa0453** | 0 | 1.2 |  |
| **Sa1732** | -0.1 | 1.2 |  |
| **Sa0302** | -0.1 | 1.2 |  |
| **Sa0881** | -0.1 | 1.2 |  |
| **Tyrs** | -0.1 | 1.2 |  |
| **Sa2106** | -0.1 | 1.2 |  |
| **Rpsh** | 0 | 1.2 |  |
| **Sa0095** | -0.1 | 1.2 |  |
| **Sa1290** | 0 | 1.2 |  |
| **Sas089** | 0.1 | 1.2 |  |
| **Opucc** | 0.1 | 1.2 |  |
| **Sa1151** | 0.1 | 1.2 |  |
| **Sa0138** | -0.1 | 1.2 |  |
| **Sa1235** | 0 | 1.2 |  |
| **Sa0174** | 0 | 1.2 |  |
| **Sa1706** | 0.1 | 1.2 |  |
| **Sa0292** | 0.1 | 1.2 |  |
| **Sa0889** | 0 | 1.2 |  |
| **Sa1375** | 0 | 1.2 |  |
| **Sa2151** | 0 | 1.2 |  |
| **Ahpf** | -0.1 | 1.1 |  |
| **Sa1220** | -0.1 | 1.1 |  |
| **Sa0880** | -0.1 | 1.1 |  |
| **Sa1369** | 0 | 1.1 |  |
| **Sa1824** | 0 | 1.1 |  |
| **Meci** | -0.8 | 1.1 |  |
| **Beta** | -0.1 | 1.1 |  |
| **Lacc** | -0.1 | 1.1 |  |
| **Ribd** | -0.1 | 1.1 |  |
| **Sa1435** | -0.1 | 1.1 |  |
| **Sa0524** | 0 | 1.1 |  |
| **Csbd** | 0 | 1.1 |  |
| **Sa1900** | 0 | 1.1 |  |
| **Sa0230** | 0 | 1.1 |  |
| **Oppb** | 0 | 1.1 |  |
| **Sa1578** | 0 | 1.1 |  |
| **Kdpc** | 0 | 1.1 |  |
| **Sa2351** | 0 | 1.1 |  |
| **Isdf** | 0 | 1.1 |  |
| **Opp-2D** | 0 | 1.1 |  |
| **Sa2350** | -0.4 | 1.1 |  |
| **Sa1360** | -0.4 | 1.1 |  |
| **Sa2092** | -0.1 | 1.1 |  |
| **Sa0025** | 0 | 1.1 |  |
| **Sa1300** | 0.1 | 1.1 |  |
| **Sa2274** | 0 | 1.1 |  |
| **Sa2447** | 0 | 1.1 |  |
| **Gyra** | 0.3 | 1.1 |  |
| **Ribc** | 0.3 | 1.1 |  |
| **Sa0468** | 0 | 1.1 |  |
| **Ctsr** | 0 | 1.1 |  |
| **Sa0699** | 0 | 1.1 |  |
| **Sa0792** | 0 | 1.1 |  |
| **Sa1621** | 0 | 1.1 |  |
| **Sa1903** | 0 | 1.1 |  |
| **Czra** | 0 | 1.1 |  |
| **Laca** | 0 | 1.1 |  |
| **Sa1799** | -0.3 | 1.1 |  |
| **Sa2473** | -0.3 | 1.1 |  |
| **Lctp** | -0.1 | 1.1 |  |
| **Sa1576** | -0.1 | 1.1 |  |
| **Sa0990** | -0.1 | 1.1 |  |
| **Sa0098** | -0.2 | 1.1 |  |
| **Sa1286** | -0.1 | 1.1 |  |
| **Sa1568** | -0.1 | 1.1 |  |
| **Sa2369** | -0.1 | 1.1 |  |
| **Sa0579** | 0 | 1.1 |  |
| **Sa1544** | -0.1 | 1.1 |  |
| **Isda** | -0.1 | 1.1 |  |
| **Sa0207** | -0.1 | 1.1 |  |
| **Sa2276** | 0 | 1.1 |  |
| **Sa2010** | 0 | 1.1 |  |
| **Icac** | 0.1 | 1.1 |  |
| **Sa0121** | 0 | 1 |  |
| **Deod** | 0 | 1 |  |
| **Sa0088** | 0 | 1 |  |
| **Sa0247** | 0 | 1 |  |
| **Sa2019** | 0 | 1 |  |
| **Sa1272** | -0.2 | 1 |  |
| **Sa1673** | -0.1 | 1 |  |
| **Sa1275** | 0 | 1 |  |
| **Muts** | -0.1 | 1 |  |
| **Murf** | -0.1 | 1 |  |
| **Sa0203** | -0.1 | 1 |  |
| **Sa0339** | 0 | 1 |  |
| **Sa0786** | 0 | 1 |  |
| **Sa1710** | 0 | 1 |  |
| **Opp-1B** | -0.1 | 1 |  |
| **Sa1198** | 0 | 1 |  |
| **Sa0621** | 0 | 1 |  |
| **Sa1007** | 0 | 1 |  |
| **Sa0193** | -0.1 | 1 |  |
| **Lig** | 0 | 1 |  |
| **Sa1173** | -0.1 | 1 |  |
| **Sa0577** | 0 | 1 |  |
| **Sa2476** | -0.1 | 1 |  |
| **Sa2004** | -0.1 | 1 |  |
| **Sa0798** | 0 | 1 |  |
| **Sa2128** | 0 | 1 |  |
| **Sas011** | 0 | 1 |  |
| **Set/06** | 0 | 1 |  |
| **Sa0516** | 0 | 1 |  |
| **Llm** | 0 | 1 |  |
| **Rsbw** | 0 | 1 |  |
| **Sa2006** | 0 | 1 |  |
| **Sa2101** | 0 | 1 |  |
| **Sa2498** | -0.1 | 1 |  |
| **Panc** | 0 | 1 |  |
| **Sa0231** | 0.1 | 1 |  |
| **Fema** | 0 | 1 |  |
| **Taga** | 0 | 1 |  |
| **Sa0631** | 0 | 1 |  |
| **Sa0780** | 0 | 1 |  |
| **Sa1419** | 0 | 1 |  |
| **Sa1715** | 0 | 1 |  |
| **Sbnc** | -0.1 | 0.9 |  |
| **Pdhc** | -0.1 | 0.9 |  |
| **Pyrc** | -0.1 | 0.9 |  |
| **Sa1132** | -0.1 | 0.9 |  |
| **Recu** | -0.1 | 0.9 |  |
| **Vals** | -0.1 | 0.9 |  |
| **Sa1593** | -0.1 | 0.9 |  |
| **Sa2299** | -0.2 | 0.9 |  |
| **Pmi** | -0.1 | 0.9 |  |
| **Sa2442** | -0.1 | 0.9 |  |
| **Sa0357** | -0.1 | 0.9 |  |
| **Sa1987** | -0.1 | 0.9 |  |
| **Gyrb** | -0.1 | 0.9 |  |
| **Capj** | -0.1 | 0.9 |  |
| **Hmra** | 0.1 | 0.9 |  |
| **Sa1004** | 0 | 0.9 |  |
| **Sa0738** | 0 | 0.9 |  |
| **Sera** | -0.1 | 0.9 |  |
| **Arab** | -0.1 | 0.9 |  |
| **Fus** | 0 | 0.9 |  |
| **Sa1430** | 0.1 | 0.9 |  |
| **Nupc** | 0 | 0.9 |  |
| **Rplk** | 0 | 0.9 |  |
| **Sa0740** | 0 | 0.9 |  |
| **Lgt** | 0 | 0.9 |  |
| **Sa1682** | 0 | 0.9 |  |
| **Sa0657** | 0 | 0.9 |  |
| **Sa1731** | 0 | 0.9 |  |
| **Mnhe** | 0 | 0.9 |  |
| **Sa0741** | -0.1 | 0.9 |  |
| **Sa0725** | 0 | 0.9 |  |
| **Sa2012** | -0.3 | 0.9 |  |
| **Sa0212** | 0 | 0.9 |  |
| **Sa1062** | 0 | 0.9 |  |
| **Lysa** | 0 | 0.9 |  |
| **Groel** | 0 | 0.9 |  |
| **Biob** | 0 | 0.9 |  |
| **Sa2241** | 0 | 0.9 |  |
| **Prop** | 0 | 0.9 |  |
| **Rela** | 0.1 | 0.9 |  |
| **Sa1221** | -0.1 | 0.9 |  |
| **Sa0341** | 0.2 | 0.9 |  |
| **Sa1277** | -0.1 | 0.8 |  |
| **Sa2395** | -0.1 | 0.8 |  |
| **Fhub** | 0 | 0.8 |  |
| **Sa2129** | -0.3 | 0.8 |  |
| **Capd** | -0.1 | 0.8 |  |
| **Sa0161** | 0 | 0.8 |  |
| **Sa0361** | 0 | 0.8 |  |
| **Sa1242** | 0 | 0.8 |  |
| **Sa2165** | 0 | 0.8 |  |
| **Rpoe** | 0 | 0.8 |  |
| **Sa0021** | 0 | 0.8 |  |
| **Sa0130** | -0.1 | 0.8 |  |
| **Rbsk** | 0 | 0.8 |  |
| **Sa0267** | 0 | 0.8 |  |
| **Sa0570** | 0 | 0.8 |  |
| **Sa0584** | 0 | 0.8 |  |
| **Sa0608** | 0 | 0.8 |  |
| **Sa0759** | 0 | 0.8 |  |
| **Gsab** | 0 | 0.8 |  |
| **Atpb** | 0 | 0.8 |  |
| **Sa1916** | -0.1 | 0.8 |  |
| **Sa2485** | 0 | 0.8 |  |
| **Sa2491** | 0 | 0.8 |  |
| **Rnpa** | 0 | 0.8 |  |
| **Sa1086** | -0.5 | 0.8 |  |
| **Sa0284** | -0.1 | 0.8 |  |
| **Sa0903** | -0.1 | 0.8 |  |
| **Sa0628** | 0.1 | 0.8 |  |
| **Prsa** | 0.1 | 0.8 |  |
| **Lpl9** | 0.1 | 0.8 |  |
| **Hlga** | -0.1 | 0.8 |  |
| **Sa1261** | 0 | 0.8 |  |
| **Sa1121** | -0.2 | 0.8 |  |
| **Sa2231** | -0.2 | 0.8 |  |
| **Sa1566** | -0.1 | 0.8 |  |
| **Sa1512** | -0.1 | 0.8 |  |
| **Rpmf** | 0 | 0.8 |  |
| **Sa1532** | 0 | 0.8 |  |
| **Capm** | 0 | 0.8 |  |
| **Sa0164** | 0 | 0.8 |  |
| **Sas009** | 0 | 0.8 |  |
| **Rpsd** | 0 | 0.8 |  |
| **Sa1612** | 0 | 0.8 |  |
| **Sa2154** | 0 | 0.8 |  |
| **Sas007** | 0 | 0.8 |  |
| **Sas008** | 0 | 0.8 |  |
| **Veg** | 0 | 0.8 |  |
| **Sas015** | 0 | 0.8 |  |
| **Rpsl** | 0 | 0.8 |  |
| **Sa0692** | 0 | 0.8 |  |
| **Sa0712** | 0 | 0.8 |  |
| **Sa0752** | 0 | 0.8 |  |
| **Sas068** | 0 | 0.8 |  |
| **Rpme** | 0 | 0.8 |  |
| **Sa1925** | 0 | 0.8 |  |
| **Sa1944** | 0 | 0.8 |  |
| **Sas069** | 0 | 0.8 |  |
| **Sas071** | 0 | 0.8 |  |
| **Sas091** | 0 | 0.8 |  |
| **Saes** | 0 | 0.8 |  |
| **Sa1833** | 0 | 0.8 |  |
| **Sa1834** | 0 | 0.8 |  |
| **Sa2433** | 0.3 | 0.8 |  |
| **Sa0324** | 0 | 0.8 |  |
| **Sa1839** | 0 | 0.8 |  |
| **Sa0013** | -0.2 | 0.8 |  |
| **Sa2302** | -0.1 | 0.8 |  |
| **Sa0140** | -0.1 | 0.8 |  |
| **Sa0255** | 0 | 0.8 |  |
| **Sa0943** | 0 | 0.8 |  |
| **Sa1548** | 0 | 0.8 |  |
| **Lsp** | 0 | 0.8 |  |
| **Sa0937** | 0 | 0.8 |  |
| **Sa0410** | 0.1 | 0.8 |  |
| **Sas063** | -0.1 | 0.8 |  |
| **Sa0399** | 0 | 0.8 |  |
| **Ftsh** | -0.1 | 0.8 |  |
| **Sa0257** | -0.1 | 0.8 |  |
| **Sa0775** | -0.1 | 0.8 |  |
| **Sa1571** | 0 | 0.8 |  |
| **Sa1988** | 0 | 0.8 |  |
| **Sa0529** | 0 | 0.8 |  |
| **Sas062** | -0.3 | 0.8 |  |
| **Agrb** | 0 | 0.8 |  |
| **Sa2133** | 0.1 | 0.8 |  |
| **Sa0964** | 0 | 0.8 |  |
| **Sa1296** | -0.1 | 0.8 |  |
| **Alss** | 0.1 | 0.8 |  |
| **Sa2455** | 0.1 | 0.8 |  |
| **Sa0956** | 0 | 0.8 |  |
| **Srrb** | -0.1 | 0.8 |  |
| **Sa0423** | -0.1 | 0.8 |  |
| **Sa2212** | -0.1 | 0.8 |  |
| **Sa1110** | 0 | 0.8 |  |
| **Sa0682** | -0.1 | 0.8 |  |
| **Sa1249** | 0.4 | 0.7 |  |
| **Sa0296** | 0 | 0.7 |  |
| **Sa1059** | 0 | 0.7 |  |
| **Sa1448** | 0 | 0.7 |  |
| **Sa1058** | -0.1 | 0.7 |  |
| **Sa1356** | 0 | 0.7 |  |
| **Sdrc** | 0 | 0.7 |  |
| **Sa0217** | 0 | 0.7 |  |
| **Sa0532** | 0 | 0.7 |  |
| **Sa0635** | 0 | 0.7 |  |
| **Sa0644** | 0 | 0.7 |  |
| **Sa0751** | 0 | 0.7 |  |
| **Sa0799** | 0 | 0.7 |  |
| **Pyrr** | 0 | 0.7 |  |
| **Sa1678** | 0 | 0.7 |  |
| **Sa1685** | 0 | 0.7 |  |
| **Sa1876** | 0 | 0.7 |  |
| **Sa1975** | 0 | 0.7 |  |
| **Sa1980** | 0 | 0.7 |  |
| **Sa2449** | 0 | 0.7 |  |
| **Icad** | 0 | 0.7 |  |
| **Sa2477** | 0 | 0.7 |  |
| **Truncated(Radc)** | -0.2 | 0.7 |  |
| **Mvak2** | 0 | 0.7 |  |
| **Sa0689** | 0 | 0.7 |  |
| **Pstb** | 0 | 0.7 |  |
| **Atph** | 0 | 0.7 |  |
| **Sa1912** | 0 | 0.7 |  |
| **Tdk** | 0 | 0.7 |  |
| **Sa2116** | 0 | 0.7 |  |
| **Sa2409** | 0 | 0.7 |  |
| **Drp35** | 0 | 0.7 |  |
| **Nrde** | -0.3 | 0.7 |  |
| **Sa0305** | -0.1 | 0.7 |  |
| **Ptaa** | -0.1 | 0.7 |  |
| **Mvaa** | 0 | 0.7 |  |
| **Sa1049** | 0 | 0.7 |  |
| **Sa1349** | -0.1 | 0.7 |  |
| **Sa2296** | -0.1 | 0.7 |  |
| **Sa0082** | -0.1 | 0.7 |  |
| **Sa1262** | -0.2 | 0.7 |  |
| **Hemy** | -0.1 | 0.7 |  |
| **Capo** | -0.1 | 0.7 |  |
| **Lpl4** | 0 | 0.7 |  |
| **Sa1632** | 0 | 0.7 |  |
| **Sa0127** | 0.1 | 0.7 |  |
| **Sa0242** | 0 | 0.7 |  |
| **Qoxb** | 0 | 0.7 |  |
| **Sa1971** | 0 | 0.7 |  |
| **Glpk** | 0 | 0.7 |  |
| **Sa2053** | -0.3 | 0.7 |  |
| **Nasd** | -0.1 | 0.7 |  |
| **Sa1008** | 0 | 0.7 |  |
| **Sa1945** | -0.3 | 0.7 |  |
| **Sa0758** | 0 | 0.7 |  |
| **Sa0215** | 0 | 0.7 |  |
| **Gercb** | 0 | 0.7 |  |
| **Metk** | 0 | 0.7 |  |
| **Glms** | 0 | 0.7 |  |
| **Args** | 0 | 0.7 |  |
| **Msrr** | 0 | 0.7 |  |
| **Sa2456** | 0 | 0.7 |  |
| **Sa2264** | 0 | 0.7 |  |
| **Dnan** | 0 | 0.7 |  |
| **Sa1418** | 0 | 0.7 |  |
| **Agrd** | 0 | 0.7 |  |
| **Sa2077** | 0 | 0.7 |  |
| **Sa2134** | 0 | 0.7 |  |
| **Sa2157** | 0 | 0.7 |  |
| **Sei** | 0.1 | 0.7 |  |
| **Fnbb** | -0.1 | 0.7 |  |
| **Sa2375** | 0.1 | 0.7 |  |
| **Sas035** | 0 | 0.7 |  |
| **Ksga** | -0.1 | 0.7 |  |
| **Citb** | -0.1 | 0.7 |  |
| **Sa2131** | 0 | 0.7 |  |
| **Sa0629** | 0 | 0.7 |  |
| **Sa1442** | 0 | 0.7 |  |
| **Sa2429** | 0 | 0.7 |  |
| **Sa0221** | 0 | 0.7 |  |
| **Sa0237** | 0 | 0.7 |  |
| **Sa0277** | 0 | 0.7 |  |
| **Sa0444** | 0 | 0.7 |  |
| **Sa0455** | 0 | 0.7 |  |
| **Nagb** | 0 | 0.7 |  |
| **Sa0536** | 0 | 0.7 |  |
| **Sas016** | 0 | 0.7 |  |
| **Sa0543** | -0.1 | 0.7 |  |
| **Sa0583** | 0 | 0.7 |  |
| **Sa0662** | 0 | 0.7 |  |
| **Sa0671** | 0 | 0.7 |  |
| **Secg** | 0 | 0.7 |  |
| **Sas020** | 0 | 0.7 |  |
| **Sa0797** | 0 | 0.7 |  |
| **Sa0800** | 0 | 0.7 |  |
| **Mnhf** | 0 | 0.7 |  |
| **Sa0914** | 0 | 0.7 |  |
| **Sa1210** | 0 | 0.7 |  |
| **Sa1217** | 0 | 0.7 |  |
| **Rpst** | 0 | 0.7 |  |
| **Hiss** | 0 | 0.7 |  |
| **Ribh** | 0 | 0.7 |  |
| **Sa1849** | 0 | 0.7 |  |
| **Sa1855** | 0 | 0.7 |  |
| **Sa1857** | 0 | 0.7 |  |
| **Sa1860** | 0 | 0.7 |  |
| **Sa1901** | 0 | 0.7 |  |
| **Atpe** | 0 | 0.7 |  |
| **Lacr** | 0 | 0.7 |  |
| **Sa2000** | 0 | 0.7 |  |
| **Sa2007** | 0 | 0.7 |  |
| **Sa2173** | 0 | 0.7 |  |
| **Sa2246** | 0 | 0.7 |  |
| **Sa2332** | 0 | 0.7 |  |
| **Sa2345** | 0 | 0.7 |  |
| **Sa2364** | 0 | 0.7 |  |
| **Sa2373** | 0 | 0.7 |  |
| **Sa2484** | 0 | 0.7 |  |
| **Sa0200** | -0.1 | 0.7 |  |
| **Ftsa** | 0 | 0.7 |  |
| **Sa1130** | 0 | 0.7 |  |
| **Sa1172** | -0.1 | 0.7 |  |
| **Sa2443** | 0 | 0.7 |  |
| **Sa1169** | -0.1 | 0.7 |  |
| **Sa2298** | -0.1 | 0.7 |  |
| **Sa1199** | -0.1 | 0.7 |  |
| **Sa2270** | -0.1 | 0.7 |  |
| **Pbpa** | -0.1 | 0.7 |  |
| **Lytn** | 0.1 | 0.6 |  |
| **Sa0329** | -0.1 | 0.6 |  |
| **Sa0368** | -0.1 | 0.6 |  |
| **Ccra** | -0.8 | 0.6 |  |
| **Sa2478** | 0 | 0.6 |  |
| **Pyrb** | 0 | 0.6 |  |
| **Dnac** | 0 | 0.6 |  |
| **Sa0615** | 0 | 0.6 |  |
| **Sa1237** | -0.1 | 0.6 |  |
| **Sa0085** | 0.1 | 0.6 |  |
| **Sa1527** | 0 | 0.6 |  |
| **Sa0647** | 0 | 0.6 |  |
| **Sa1000** | 0 | 0.6 |  |
| **Sa0588** | 0 | 0.6 |  |
| **Nrdf** | 0 | 0.6 |  |
| **Div1b** | 0 | 0.6 |  |
| **Sa0343** | 0 | 0.6 |  |
| **Sa0351** | -0.1 | 0.6 |  |
| **Ruvb** | 0 | 0.6 |  |
| **Sa1898** | 0 | 0.6 |  |
| **Sa2108** | 0 | 0.6 |  |
| **Sers** | 0 | 0.6 |  |
| **Sa0192** | 0 | 0.6 |  |
| **Sa0205** | 0 | 0.6 |  |
| **Sa0587** | -0.1 | 0.6 |  |
| **Sa0815** | 0 | 0.6 |  |
| **Sa0939** | 0 | 0.6 |  |
| **Sa1973** | 0 | 0.6 |  |
| **Sa2479** | 0 | 0.6 |  |
| **Folb** | 0 | 0.6 |  |
| **Sa0515** | 0 | 0.6 |  |
| **Sa1079** | 0 | 0.6 |  |
| **Sa1976** | 0 | 0.6 |  |
| **Sa2382** | 0 | 0.6 |  |
| **Cspc** | -0.1 | 0.6 |  |
| **Phoh** | 0 | 0.6 |  |
| **Citg** | 0 | 0.6 |  |
| **Sa2247** | 0 | 0.6 |  |
| **Sa1738** | -0.1 | 0.6 |  |
| **Sa0867** | 0 | 0.6 |  |
| **Sa1607** | -0.1 | 0.6 |  |
| **Sa2327** | -0.1 | 0.6 |  |
| **Glpf** | -0.1 | 0.6 |  |
| **Sa1284** | -0.1 | 0.6 |  |
| **Sa1490** | -0.1 | 0.6 |  |
| **Sa0427** | 0 | 0.6 |  |
| **Sa1108** | 0 | 0.6 |  |
| **Sa0567** | 0 | 0.6 |  |
| **Sa0517** | 0 | 0.6 |  |
| **Plc** | 0 | 0.6 |  |
| **Sa1989** | -0.1 | 0.6 |  |
| **Sa2259** | 0 | 0.6 |  |
| **Sa1250** | 0 | 0.6 |  |
| **Sa1600** | 0 | 0.6 |  |
| **Vras** | 0 | 0.6 |  |
| **Cina** | 0 | 0.6 |  |
| **Sa1266** | 0 | 0.6 |  |
| **Sa0123** | 0 | 0.6 |  |
| **Sa1294** | 0 | 0.6 |  |
| **Sa0932** | 0 | 0.6 |  |
| **Sa2059** | 0.1 | 0.6 |  |
| **Kdpd(Sccmec)** | -0.8 | 0.6 |  |
| **Sa0672** | -0.1 | 0.6 |  |
| **Sa2216** | -0.4 | 0.6 |  |
| **Sart** | 0 | 0.6 |  |
| **Sa1790** | -0.1 | 0.6 |  |
| **Panb** | 0 | 0.6 |  |
| **Sa2163** | 0 | 0.6 |  |
| **Sa1331** | -0.1 | 0.6 |  |
| **Isdb** | -0.1 | 0.6 |  |
| **Sa2228** | -0.1 | 0.6 |  |
| **Ddh** | 0 | 0.6 |  |
| **Tkt** | 0 | 0.6 |  |
| **Sa1432** | 0 | 0.6 |  |
| **Mvas** | 0 | 0.6 |  |
| **Sa0314** | 0 | 0.6 |  |
| **Sa2103** | 0 | 0.6 |  |
| **Sa1640** | -0.1 | 0.6 |  |
| **Murg** | 0 | 0.6 |  |
| **Sa0381** | 0 | 0.6 |  |
| **Sas014** | 0 | 0.6 |  |
| **Dapd** | 0 | 0.6 |  |
| **Sa1450** | 0 | 0.6 |  |
| **Rplx** | 0 | 0.6 |  |
| **Sa2062** | 0 | 0.6 |  |
| **Ureb** | 0 | 0.6 |  |
| **Sa2321** | 0 | 0.6 |  |
| **Sa2421** | 0 | 0.6 |  |
| **Sa0607** | 0 | 0.6 |  |
| **Sa0003** | 0 | 0.6 |  |
| **Lrga** | 0 | 0.6 |  |
| **Sa0278** | 0 | 0.6 |  |
| **Sa0313** | 0 | 0.6 |  |
| **Sa0446** | 0 | 0.6 |  |
| **Sa0464** | 0 | 0.6 |  |
| **Sa0491** | 0 | 0.6 |  |
| **Rpsg** | 0 | 0.6 |  |
| **Ung** | -0.1 | 0.6 |  |
| **Sa0550** | 0 | 0.6 |  |
| **Tagd** | 0 | 0.6 |  |
| **Sas017** | 0 | 0.6 |  |
| **Sa0612** | 0 | 0.6 |  |
| **Sa0614** | 0 | 0.6 |  |
| **Sa0666** | 0 | 0.6 |  |
| **Prfb** | 0 | 0.6 |  |
| **Sa0746** | 0 | 0.6 |  |
| **Sa0756** | 0 | 0.6 |  |
| **Sa0760** | 0 | 0.6 |  |
| **Dltc** | 0 | 0.6 |  |
| **Sa0805** | 0 | 0.6 |  |
| **Comeb** | 0 | 0.6 |  |
| **Sgta** | -0.1 | 0.6 |  |
| **Sa1663** | 0 | 0.6 |  |
| **Sas053** | -0.1 | 0.6 |  |
| **Sa1671** | 0 | 0.6 |  |
| **Sa1689** | 0 | 0.6 |  |
| **Sas067** | 0 | 0.6 |  |
| **Dpj** | 0 | 0.6 |  |
| **Dps** | 0 | 0.6 |  |
| **Sas073** | 0 | 0.6 |  |
| **Sas074** | 0 | 0.6 |  |
| **Sa1985** | 0 | 0.6 |  |
| **Rpsi** | 0 | 0.6 |  |
| **Sa2090** | 0 | 0.6 |  |
| **Sa2245** | 0 | 0.6 |  |
| **Cspb** | 0 | 0.6 |  |
| **Sa2144** | 0 | 0.6 |  |
| **Sa1013** | 0 | 0.6 |  |
| **Sa1158** | 0 | 0.6 |  |
| **Pyraa** | 0 | 0.5 |  |
| **Sarr** | 0 | 0.5 |  |
| **Sas028** | 0 | 0.5 |  |
| **Sa1030** | -0.1 | 0.5 |  |
| **Sa1056** | 0 | 0.5 |  |
| **Sa1304** | 0 | 0.5 |  |
| **Sa2219** | 0 | 0.5 |  |
| **Vrad** | 0 | 0.5 |  |
| **Sa0511** | 0 | 0.5 |  |
| **Sa1230** | -0.1 | 0.5 |  |
| **Sa1370** | 0 | 0.5 |  |
| **Hisi** | 0 | 0.5 |  |
| **Sa0176** | -0.1 | 0.5 |  |
| **Sa0377** | 0 | 0.5 |  |
| **Sa0526** | -0.1 | 0.5 |  |
| **Sa0561** | 0 | 0.5 |  |
| **Tagx** | 0 | 0.5 |  |
| **Sa0755** | 0 | 0.5 |  |
| **Pcp** | 0 | 0.5 |  |
| **Sa0837** | -0.6 | 0.5 |  |
| **Hrca** | -0.1 | 0.5 |  |
| **Opp-1F** | 0 | 0.5 |  |
| **Sa0355** | 0 | 0.5 |  |
| **Sa0445** | 0 | 0.5 |  |
| **Sa0806** | 0 | 0.5 |  |
| **Bmfbb** | 0 | 0.5 |  |
| **Sa0624** | 0 | 0.5 |  |
| **Sa0844** | 0 | 0.5 |  |
| **Sa1372** | 0 | 0.5 |  |
| **Leua** | 0 | 0.5 |  |
| **Sa2204** | 0 | 0.5 |  |
| **Sa2488** | 0 | 0.5 |  |
| **Sa0863** | -0.3 | 0.5 |  |
| **Sa0311** | -0.1 | 0.5 |  |
| **Sa0968** | 0 | 0.5 |  |
| **Isdc** | 0 | 0.5 |  |
| **Sa1131** | -0.1 | 0.5 |  |
| **Sa1311** | 0 | 0.5 |  |
| **Sa0322** | 0 | 0.5 |  |
| **Sa2413** | -0.1 | 0.5 |  |
| **Sa0235** | 0 | 0.5 |  |
| **Sas004** | 0 | 0.5 |  |
| **Sa0160** | 0 | 0.5 |  |
| **Rpsf** | 0 | 0.5 |  |
| **Sa0360** | 0 | 0.5 |  |
| **Sa0364** | 0 | 0.5 |  |
| **Sa0372** | 0 | 0.5 |  |
| **Sa0816** | 0 | 0.5 |  |
| **Sas026** | 0 | 0.5 |  |
| **Sa1443** | 0 | 0.5 |  |
| **Sas049** | 0 | 0.5 |  |
| **Sa1464** | 0 | 0.5 |  |
| **Sa1472** | 0 | 0.5 |  |
| **Rplu** | 0 | 0.5 |  |
| **Sa1543** | 0 | 0.5 |  |
| **Sa1696** | 0 | 0.5 |  |
| **Sa1723** | 0 | 0.5 |  |
| **Rpsm** | 0 | 0.5 |  |
| **Rpmj** | 0 | 0.5 |  |
| **Sas082** | 0 | 0.5 |  |
| **Sa2309** | 0 | 0.5 |  |
| **Sa2331** | 0 | 0.5 |  |
| **Sa1814** | -0.1 | 0.5 |  |
| **Sa0523** | 0 | 0.5 |  |
| **Sa1365** | -0.1 | 0.5 |  |
| **Ptsi** | -0.1 | 0.5 |  |
| **Sa0470** | -0.2 | 0.5 |  |
| **Sa0606** | -0.1 | 0.5 |  |
| **Phet** | -0.1 | 0.5 |  |
| **Nuc** | -0.1 | 0.5 |  |
| **Hysa** | -0.1 | 0.5 |  |
| **Sa1815** | -0.1 | 0.5 |  |
| **Sa1136** | 0.1 | 0.5 |  |
| **Ebha** | 0 | 0.5 |  |
| **Sa1415** | 0 | 0.5 |  |
| **Sa1820** | 0 | 0.5 |  |
| **Sa1825** | 0 | 0.5 |  |
| **Hsdm** | 0 | 0.5 |  |
| **Clpy** | 0 | 0.5 |  |
| **Ebhb** | 0 | 0.5 |  |
| **Sa2238** | 0 | 0.5 |  |
| **Sa0059** | 0 | 0.5 |  |
| **Sa0173** | 0 | 0.5 |  |
| **Rpla** | 0 | 0.5 |  |
| **Rpso** | 0 | 0.5 |  |
| **Sa1552** | 0 | 0.5 |  |
| **Sa1577** | 0.2 | 0.5 |  |
| **Sa1599** | 0 | 0.5 |  |
| **Sec3** | 0 | 0.5 |  |
| **Sa1828** | 0 | 0.5 |  |
| **Sa1831** | 0 | 0.5 |  |
| **Rho** | 0 | 0.5 |  |
| **Sa2275** | 0 | 0.5 |  |
| **Ptsg** | 0 | 0.5 |  |
| **Meca** | 0 | 0.5 |  |
| **Mecr1** | 0 | 0.5 |  |
| **Sa0100** | 0.1 | 0.5 |  |
| **Spa** | 0 | 0.5 |  |
| **Sbna** | 0 | 0.5 |  |
| **Sa0125** | 0.2 | 0.5 |  |
| **Capb** | 0 | 0.5 |  |
| **Sa0169** | 0.2 | 0.5 |  |
| **Sa0220** | 0 | 0.5 |  |
| **Sa0225** | 0 | 0.5 |  |
| **Sa0239** | 0 | 0.5 |  |
| **Sa0297** | 0 | 0.5 |  |
| **Sa0323** | 0.1 | 0.5 |  |
| **Sa0326** | 0 | 0.5 |  |
| **Sa0362** | 0 | 0.5 |  |
| **Pbux** | 0 | 0.5 |  |
| **Gltb** | 0 | 0.5 |  |
| **Gltd** | 0 | 0.5 |  |
| **Trep** | 0 | 0.5 |  |
| **Holb** | 0 | 0.5 |  |
| **Sa0449** | 0 | 0.5 |  |
| **Mfd** | 0.3 | 0.5 |  |
| **Rplj** | 0 | 0.5 |  |
| **Sa0522** | 0 | 0.5 |  |
| **Adh1** | 0 | 0.5 |  |
| **Sa0569** | 0 | 0.5 |  |
| **Sa0581** | 0 | 0.5 |  |
| **Tagg** | 0 | 0.5 |  |
| **Sa0639** | 0 | 0.5 |  |
| **Sa0642** | 0 | 0.5 |  |
| **Sa0659** | 0 | 0.5 |  |
| **Sa0669** | 0 | 0.5 |  |
| **Uvra** | 0 | 0.5 |  |
| **Pgm** | 0 | 0.5 |  |
| **Sa0743** | 0 | 0.5 |  |
| **Sa0750** | 0.3 | 0.5 |  |
| **Sa0769** | 0 | 0.5 |  |
| **Dltd** | 0 | 0.5 |  |
| **Sa0859** | 0.4 | 0.5 |  |
| **Sa0938** | 0 | 0.5 |  |
| **Sa0957** | 0 | 0.5 |  |
| **Sa0962** | 0 | 0.5 |  |
| **Muts2** | 0 | 0.5 |  |
| **Argf** | 0.3 | 0.5 |  |
| **Sa1014** | 0 | 0.5 |  |
| **Spoiiie** | 0 | 0.5 |  |
| **Sa1120** | 0 | 0.5 |  |
| **Sa1155** | 0 | 0.5 |  |
| **Parc** | 0.3 | 0.5 |  |
| **Sa1231** | 0 | 0.5 |  |
| **Sa1238** | 0 | 0.5 |  |
| **Odha** | 0 | 0.5 |  |
| **Ctpa** | 0 | 0.5 |  |
| **Sa1258** | 0 | 0.5 |  |
| **Sa1264** | 0 | 0.5 |  |
| **Pbp2** | 0 | 0.5 |  |
| **Sa1295** | 0 | 0.5 |  |
| **Ebps** | 0 | 0.5 |  |
| **Sa1317** | 0 | 0.5 |  |
| **Mala** | 0 | 0.5 |  |
| **Sa1387** | 0 | 0.5 |  |
| **Siga** | 0 | 0.5 |  |
| **Glys** | 0 | 0.5 |  |
| **Sa1403** | 0 | 0.5 |  |
| **Sa1405** | 0 | 0.5 |  |
| **Lepa** | 0 | 0.5 |  |
| **Sa1454** | 0.3 | 0.5 |  |
| **Thrs** | 0 | 0.5 |  |
| **Ald** | 0 | 0.5 |  |
| **Sa1537** | 0 | 0.5 |  |
| **Sa1539** | 0 | 0.5 |  |
| **Sa1542** | 0 | 0.5 |  |
| **Acuc** | 0 | 0.5 |  |
| **Sa1569** | 0 | 0.5 |  |
| **Sa1676** | 0 | 0.5 |  |
| **Sa1716** | 0 | 0.5 |  |
| **Sel** | 0 | 0.5 |  |
| **Sa1818** | 0 | 0.5 |  |
| **Tst** | 0 | 0.5 |  |
| **Sa1821** | 0 | 0.5 |  |
| **Sa1822** | 0 | 0.5 |  |
| **Sa1826** | 0 | 0.5 |  |
| **Sa1827** | 0 | 0.5 |  |
| **Sa1829** | 0 | 0.5 |  |
| **Sa1830** | 0 | 0.5 |  |
| **Sa1832** | 0 | 0.5 |  |
| **Sa1854** | 0 | 0.5 |  |
| **Ilvb** | 0 | 0.5 |  |
| **Ilva** | 0 | 0.5 |  |
| **Sa1966** | 0 | 0.5 |  |
| **Lace** | 0 | 0.5 |  |
| **Sa2021** | 0 | 0.5 |  |
| **Rpsq** | 0 | 0.5 |  |
| **Fni** | 0 | 0.5 |  |
| **Tcaa** | 0 | 0.5 |  |
| **Sa2156** | 0 | 0.5 |  |
| **Narh** | 0 | 0.5 |  |
| **Sa2232** | 0 | 0.5 |  |
| **Fnb** | 0 | 0.5 |  |
| **Sa2301** | 0 | 0.5 |  |
| **Sa2381** | 0 | 0.5 |  |
| **Sa2432** | 0 | 0.5 |  |
| **Sa2445** | 0 | 0.5 |  |
| **Sa2446** | 0 | 0.5 |  |
| **Sa2475** | 0 | 0.5 |  |
| **Sa0061** | -0.5 | 0.5 |  |
| **Ctra** | 0 | 0.5 |  |
| **Sa0283** | 0 | 0.5 |  |
| **Citz** | 0 | 0.5 |  |
| **Clfb** | -0.3 | 0.5 |  |
| **Sa2319** | 0.1 | 0.5 |  |
| **Sa2177** | -0.3 | 0.5 |  |
| **Sa1447** | 0 | 0.5 |  |
| **Sa0674** | 0 | 0.5 |  |
| **Sa0244** | -0.1 | 0.5 |  |
| **Leuc** | -0.1 | 0.5 |  |
| **Sas001** | 0 | 0.5 |  |
| **Rpli** | 0 | 0.5 |  |
| **Sa0213** | -0.1 | 0.5 |  |
| **Sa0236** | 0 | 0.5 |  |
| **Lrgb** | 0 | 0.5 |  |
| **Sa0279** | 0 | 0.5 |  |
| **Pth** | 0 | 0.5 |  |
| **Sa0465** | 0 | 0.5 |  |
| **Sa0481** | 0 | 0.5 |  |
| **Sece** | 0 | 0.5 |  |
| **Sa0514** | 0 | 0.5 |  |
| **Sa0546** | 0 | 0.5 |  |
| **Sa0559** | 0 | 0.5 |  |
| **Sa0576** | 0 | 0.5 |  |
| **Sa0651** | 0 | 0.5 |  |
| **Sa0683** | 0 | 0.5 |  |
| **Sa0693** | 0 | 0.5 |  |
| **Sa0722** | 0 | 0.5 |  |
| **Sa0791** | 0 | 0.5 |  |
| **Sas024** | 0 | 0.5 |  |
| **Sas027** | 0 | 0.5 |  |
| **Purc** | 0 | 0.5 |  |
| **Rbfa** | 0 | 0.5 |  |
| **Sa1185** | 0 | 0.5 |  |
| **Sa1186** | 0 | 0.5 |  |
| **Cspa** | 0 | 0.5 |  |
| **Fer** | 0 | 0.5 |  |
| **Sa1316** | 0 | 0.5 |  |
| **Accb** | 0 | 0.5 |  |
| **Sa1441** | 0 | 0.5 |  |
| **Sa1712** | -0.1 | 0.5 |  |
| **Rsbv** | 0 | 0.5 |  |
| **Sa1878** | -0.1 | 0.5 |  |
| **Atpf** | 0 | 0.5 |  |
| **Upp** | 0 | 0.5 |  |
| **Sa1917** | 0 | 0.5 |  |
| **Prfa** | 0 | 0.5 |  |
| **Sa1933** | 0 | 0.5 |  |
| **Sa1937** | 0 | 0.5 |  |
| **Asp23** | 0 | 0.5 |  |
| **Sas075** | 0 | 0.5 |  |
| **Rpsc** | 0 | 0.5 |  |
| **Sa2076** | 0 | 0.5 |  |
| **Sa2097** | 0 | 0.5 |  |
| **Sa2118** | 0 | 0.5 |  |
| **Sa2135** | 0 | 0.5 |  |
| **Sa2178** | 0 | 0.5 |  |
| **Sa2211** | 0 | 0.5 |  |
| **Sa2258** | 0 | 0.5 |  |
| **Sa2340** | 0 | 0.5 |  |
| **Sa2362** | 0 | 0.5 |  |
| **Sa2398** | 0 | 0.5 |  |
| **Sa2450** | 0 | 0.5 |  |
| **Sa2454** | 0 | 0.5 |  |
| **Sa2495** | 0 | 0.5 |  |
| **Rpmh** | 0 | 0.5 |  |
| **Agrc** | 0 | 0.5 |  |
| **Sa0234** | 0 | 0.5 |  |
| **Guaa** | 0 | 0.5 |  |
| **Sa1662** | 0 | 0.5 |  |
| **Spovg** | 0 | 0.5 |  |
| **Sa0626** | 0 | 0.5 |  |
| **Sa1982** | 0 | 0.5 |  |
| **Sa2119** | -0.1 | 0.5 |  |
| **Sa2354** | 0 | 0.5 |  |
| **Sa1020** | -0.1 | 0.5 |  |
| **Sa1355** | 0 | 0.5 |  |
| **Sa2240** | 0 | 0.5 |  |
| **Sa2448** | 0 | 0.5 |  |
| **Sa0664** | 0 | 0.5 |  |
| **Sas045** | 0 | 0.5 |  |
| **Adab** | 0 | 0.5 |  |
| **Mnhd** | 0 | 0.5 |  |
| **Sa0949** | 0 | 0.5 |  |
| **Sa1254** | 0 | 0.5 |  |
| **Ndk** | 0 | 0.5 |  |
| **Thie** | 0 | 0.5 |  |
| **Lacd** | -0.1 | 0.5 |  |
| **Sa0124** | 0 | 0.5 |  |
| **Sa0476** | 0 | 0.5 |  |
| **Sa0572** | 0 | 0.5 |  |
| **Sas029** | 0 | 0.5 |  |
| **Sa0927** | 0 | 0.5 |  |
| **Xerd** | -0.1 | 0.5 |  |
| **Sa1711** | 0 | 0.5 |  |
| **Sa1742** | 0 | 0.5 |  |
| **Sas077** | 0 | 0.5 |  |
| **Rpoa** | 0 | 0.5 |  |
| **Sa1153** | 0 | 0.5 |  |
| **Dnak** | 0 | 0.4 |  |
| **Ansa** | -0.2 | 0.4 |  |
| **Sbng** | 0 | 0.4 |  |
| **Sa0864** | 0 | 0.4 |  |
| **Argj** | -0.1 | 0.4 |  |
| **Sa0856** | 0 | 0.4 |  |
| **Sa1384** | 0 | 0.4 |  |
| **Udk** | 0 | 0.4 |  |
| **Sa1416** | 0 | 0.4 |  |
| **Ilve** | 0 | 0.4 |  |
| **Oppb(Truncated)** | 0 | 0.4 |  |
| **Sa2283** | -0.1 | 0.4 |  |
| **Sa0434** | 0 | 0.4 |  |
| **Pdhd** | 0 | 0.4 |  |
| **Sbnf** | 0 | 0.4 |  |
| **Glnr** | 0 | 0.4 |  |
| **Tnpc** | -0.4 | 0.4 |  |
| **Fhug** | -0.1 | 0.4 |  |
| **Sa0778** | -0.1 | 0.4 |  |
| **Thrb** | 0 | 0.4 |  |
| **Kata** | -0.1 | 0.4 |  |
| **Cbf1** | 0 | 0.4 |  |
| **Sa0888** | 0 | 0.4 |  |
| **Lpl7** | -0.1 | 0.4 |  |
| **Sa0739** | 0 | 0.4 |  |
| **Sa1152** | -0.3 | 0.4 |  |
| **Sa1005** | 0 | 0.4 |  |
| **Sa1003** | 0 | 0.4 |  |
| **Sas056** | 0 | 0.4 |  |
| **Gltt** | 0 | 0.4 |  |
| **Sa0415** | 0 | 0.4 |  |
| **Sa0907** | 0 | 0.4 |  |
| **Sa0188** | 0 | 0.4 |  |
| **Sa0335** | 0 | 0.4 |  |
| **Sa0336** | 0 | 0.4 |  |
| **Sa0101** | -0.1 | 0.4 |  |
| **Sa0300** | 0 | 0.4 |  |
| **Ssb** | 0 | 0.4 |  |
| **Sa0359** | 0 | 0.4 |  |
| **Sa0363** | 0 | 0.4 |  |
| **Sa0414** | 0 | 0.4 |  |
| **Mnhb** | 0 | 0.4 |  |
| **Spsa** | -0.1 | 0.4 |  |
| **Sa0910** | 0 | 0.4 |  |
| **Ptsh** | 0 | 0.4 |  |
| **Sa1423** | 0 | 0.4 |  |
| **Sa1445** | 0 | 0.4 |  |
| **Sa1610** | 0 | 0.4 |  |
| **Sa1694** | 0 | 0.4 |  |
| **Sa1698** | 0 | 0.4 |  |
| **Sa1717** | 0 | 0.4 |  |
| **Infa** | 0 | 0.4 |  |
| **Adk** | 0 | 0.4 |  |
| **Moad** | 0 | 0.4 |  |
| **Modc** | 0 | 0.4 |  |
| **Sas083** | -0.1 | 0.4 |  |
| **Nase** | 0 | 0.4 |  |
| **Sa2193** | 0 | 0.4 |  |
| **Sa2198** | 0 | 0.4 |  |
| **Sa2201** | 0 | 0.4 |  |
| **Sa2308** | 0 | 0.4 |  |
| **Sas088** | -0.1 | 0.4 |  |
| **Sa2338** | 0 | 0.4 |  |
| **Sa2343** | 0 | 0.4 |  |
| **Sa2360** | 0 | 0.4 |  |
| **Sa2361** | 0 | 0.4 |  |
| **Sa2376** | 0 | 0.4 |  |
| **Sa2407** | 0 | 0.4 |  |
| **Sa2230** | 0 | 0.4 |  |
| **Sa0553** | 0 | 0.4 |  |
| **Sa1313** | 0 | 0.4 |  |
| **Sa0871** | -0.1 | 0.4 |  |
| **Sa0718** | 0 | 0.4 |  |
| **Lpl8** | -0.1 | 0.4 |  |
| **Sa2282** | -0.1 | 0.4 |  |
| **Sa2486** | 0 | 0.4 |  |
| **Sa1368** | 0 | 0.4 |  |
| **Hemh** | 0 | 0.4 |  |
| **Lipa** | 0 | 0.4 |  |
| **Sa1015** | 0 | 0.4 |  |
| **Sa2469** | 0 | 0.4 |  |
| **Sa0010** | -0.1 | 0.4 |  |
| **Sa0105** | 0 | 0.4 |  |
| **Sas013** | 0 | 0.4 |  |
| **Cyse** | 0 | 0.4 |  |
| **Sa0618** | 0 | 0.4 |  |
| **Sa0782** | -0.1 | 0.4 |  |
| **Sspb** | 0 | 0.4 |  |
| **Sa1979** | 0 | 0.4 |  |
| **Nrga** | -0.1 | 0.4 |  |
| **Sa0011** | 0 | 0.4 |  |
| **Sa0416** | -0.1 | 0.4 |  |
| **Frub** | 0 | 0.4 |  |
| **Sa1187** | 0 | 0.4 |  |
| **Sa1326** | 0 | 0.4 |  |
| **Sa1692** | 0 | 0.4 |  |
| **Sa1974** | 0 | 0.4 |  |
| **Sa2140** | 0 | 0.4 |  |
| **Alst** | 0 | 0.4 |  |
| **Sa0840** | 0 | 0.4 |  |
| **Sa0135** | -0.1 | 0.4 |  |
| **Sa0037** | -0.1 | 0.4 |  |
| **Sa0194** | 0 | 0.4 |  |
| **Sa0450** | 0 | 0.4 |  |
| **Sa0580** | 0 | 0.4 |  |
| **Sa0632** | 0 | 0.4 |  |
| **Sa0695** | 0 | 0.4 |  |
| **Sa0734** | -0.1 | 0.4 |  |
| **Purb** | 0 | 0.4 |  |
| **Fbaa** | 0 | 0.4 |  |
| **Sa2113** | 0 | 0.4 |  |
| **Pnp** | 0 | 0.4 |  |
| **Capg** | 0 | 0.4 |  |
| **Sa0266** | 0 | 0.4 |  |
| **Folk** | 0 | 0.4 |  |
| **Sa0489** | 0 | 0.4 |  |
| **Sa0507** | -0.1 | 0.4 |  |
| **Sa0509** | 0 | 0.4 |  |
| **Sa0542** | 0 | 0.4 |  |
| **Sas025** | 0 | 0.4 |  |
| **Sa0947** | 0 | 0.4 |  |
| **Sa1057** | 0 | 0.4 |  |
| **Truncated-Arlr** | 0 | 0.4 |  |
| **Sa1340** | 0 | 0.4 |  |
| **Sa1353** | 0 | 0.4 |  |
| **Sa1362** | -0.1 | 0.4 |  |
| **Sa1560** | 0 | 0.4 |  |
| **Sa1892** | -0.1 | 0.4 |  |
| **Luxs** | 0 | 0.4 |  |
| **Moba** | 0 | 0.4 |  |
| **Sa0020** | 0 | 0.4 |  |
| **Sa0273** | 0 | 0.4 |  |
| **Sa0477** | 0 | 0.4 |  |
| **Sa0492** | 0 | 0.4 |  |
| **Sa0611** | 0 | 0.4 |  |
| **Sa0633** | 0 | 0.4 |  |
| **Sa0670** | -0.1 | 0.4 |  |
| **Nrdi** | 0 | 0.4 |  |
| **Sa0690** | 0 | 0.4 |  |
| **Sa0704** | 0 | 0.4 |  |
| **Sa0776** | 0 | 0.4 |  |
| **Sa1666** | 0 | 0.4 |  |
| **Groes** | 0 | 0.4 |  |
| **Sa1856** | 0 | 0.4 |  |
| **Leud** | 0 | 0.4 |  |
| **Sa1873** | 0 | 0.4 |  |
| **Sa1890** | 0 | 0.4 |  |
| **Thid** | 0 | 0.4 |  |
| **Sa1899** | 0 | 0.4 |  |
| **Sa1934** | 0 | 0.4 |  |
| **Opucd** | 0 | 0.4 |  |
| **Hisg** | -0.1 | 0.4 |  |
| **Opp-1A** | -0.2 | 0.4 |  |
| **Sa0895** | 0 | 0.4 |  |
| **Pola** | -0.1 | 0.4 |  |
| **Hutu** | -0.1 | 0.4 |  |
| **Sa2303** | -0.1 | 0.4 |  |
| **Vraa** | -0.1 | 0.4 |  |
| **Pcra** | 0 | 0.4 |  |
| **Sa2292** | -0.1 | 0.4 |  |
| **Sa1462** | -0.1 | 0.4 |  |
| **Gapr** | 0 | 0.4 |  |
| **Set/13** | 0 | 0.4 |  |
| **Sa1392** | 0 | 0.4 |  |
| **Roca** | 0 | 0.4 |  |
| **Sa0781** | 0 | 0.4 |  |
| **Sa1271** | 0 | 0.4 |  |
| **Mobb** | 0 | 0.4 |  |
| **Set/15** | 0 | 0.4 |  |
| **Tag** | 0 | 0.4 |  |
| **Sa0167** | -0.2 | 0.4 |  |
| **Cysm** | 0 | 0.4 |  |
| **Sa1345** | 0 | 0.4 |  |
| **Sa0347** | -0.1 | 0.4 |  |
| **Sa0893** | 0 | 0.4 |  |
| **Sa0966** | 0 | 0.4 |  |
| **Sa1279** | 0 | 0.4 |  |
| **Sa1705** | -0.1 | 0.4 |  |
| **Sa1263** | 0 | 0.4 |  |
| **Sa2050** | -0.1 | 0.4 |  |
| **Sa1336** | 0 | 0.4 |  |
| **Quea** | 0 | 0.4 |  |
| **Nusa** | -0.1 | 0.4 |  |
| **Sa1500** | 0 | 0.4 |  |
| **Sa1514** | -0.1 | 0.4 |  |
| **Sa0227** | 0 | 0.4 |  |
| **Sa0024** | -0.2 | 0.4 |  |
| **Sa1270** | -0.1 | 0.3 |  |
| **Sa1787** | -0.3 | 0.3 |  |
| **Sa0191** | -0.3 | 0.3 |  |
| **Lip** | 0 | 0.3 |  |
| **Sa0627** | 0 | 0.3 |  |
| **Sa1981** | -0.1 | 0.3 |  |
| **Sa1292** | 0 | 0.3 |  |
| **Sa0841** | 0 | 0.3 |  |
| **Sas002** | -0.5 | 0.3 |  |
| **Sa0210** | -0.3 | 0.3 |  |
| **Sas006** | 0 | 0.3 |  |
| **Sa1002** | 0 | 0.3 |  |
| **Pyka** | 0 | 0.3 |  |
| **Purk** | 0 | 0.3 |  |
| **Sa1224** | 0 | 0.3 |  |
| **Sa1699** | 0 | 0.3 |  |
| **Sa0378** | 0 | 0.3 |  |
| **Sa0084** | 0 | 0.3 |  |
| **Sirb** | 0 | 0.3 |  |
| **Sa0204** | 0 | 0.3 |  |
| **Sa0447** | 0 | 0.3 |  |
| **Sa0467** | 0 | 0.3 |  |
| **Sa0525** | 0 | 0.3 |  |
| **Sa0620** | 0 | 0.3 |  |
| **Sa0679** | 0 | 0.3 |  |
| **Sa0873** | 0 | 0.3 |  |
| **Sa0885** | 0 | 0.3 |  |
| **Pgsa** | 0 | 0.3 |  |
| **Trpd** | -0.1 | 0.3 |  |
| **Scrb** | -0.2 | 0.3 |  |
| **Sa2078** | 0 | 0.3 |  |
| **Sa0902** | 0 | 0.3 |  |
| **Sa2310** | 0 | 0.3 |  |
| **Ant(9)** | -0.4 | 0.3 |  |
| **Sa0099** | 0 | 0.3 |  |
| **Sa1649** | 0 | 0.3 |  |
| **Sa2091** | 0 | 0.3 |  |
| **Sa0882** | -0.3 | 0.3 |  |
| **Sa1939** | 0 | 0.3 |  |
| **Seg** | 0 | 0.3 |  |
| **Sas058** | -0.1 | 0.3 |  |
| **Sa0274** | 0 | 0.3 |  |
| **Clpp** | 0 | 0.3 |  |
| **Sa1022** | -0.1 | 0.3 |  |
| **Sa0530** | 0 | 0.3 |  |
| **Sbni** | 0 | 0.3 |  |
| **Sa0240** | 0 | 0.3 |  |
| **Sa0245** | 0 | 0.3 |  |
| **Set/14** | 0 | 0.3 |  |
| **Pta** | 0 | 0.3 |  |
| **Sa0575** | 0 | 0.3 |  |
| **Mray** | 0 | 0.3 |  |
| **Apt** | 0 | 0.3 |  |
| **Alr** | -0.1 | 0.3 |  |
| **Sa2009** | 0 | 0.3 |  |
| **Moda** | 0 | 0.3 |  |
| **Nark** | 0 | 0.3 |  |
| **Sa2181** | 0 | 0.3 |  |
| **Sa2225** | 0 | 0.3 |  |
| **Sa2487** | 0 | 0.3 |  |
| **Hlgb** | 0 | 0.3 |  |
| **Capa** | 0 | 0.3 |  |
| **Sa1602** | 0 | 0.3 |  |
| **Sa1734** | 0 | 0.3 |  |
| **Sa2054** | 0 | 0.3 |  |
| **Sa2159** | -0.1 | 0.3 |  |
| **Sa0132** | 0 | 0.3 |  |
| **Rnc** | 0 | 0.3 |  |
| **Sa2183** | 0 | 0.3 |  |
| **Prs** | 0 | 0.3 |  |
| **Sa0636** | -0.2 | 0.3 |  |
| **Sa0777** | 0 | 0.3 |  |
| **Sas043** | 0 | 0.3 |  |
| **Sa1219** | 0 | 0.3 |  |
| **Sa1690** | 0 | 0.3 |  |
| **Hisb** | 0 | 0.3 |  |
| **Sa0757** | 0 | 0.3 |  |
| **Sa1942** | 0 | 0.3 |  |
| **Sa2496** | 0 | 0.3 |  |
| **Sa0539** | 0 | 0.3 |  |
| **Menb** | 0 | 0.3 |  |
| **Rot** | 0 | 0.3 |  |
| **Ureg** | 0 | 0.3 |  |
| **Sa0086** | 0 | 0.3 |  |
| **Sodm** | 0 | 0.3 |  |
| **Sas012** | 0 | 0.3 |  |
| **Sa0897** | 0 | 0.3 |  |
| **Trxa** | 0 | 0.3 |  |
| **Sa1033** | 0 | 0.3 |  |
| **Rpmg** | 0 | 0.3 |  |
| **Sa1426** | 0 | 0.3 |  |
| **Sa1444** | -0.1 | 0.3 |  |
| **Sas050** | 0 | 0.3 |  |
| **Sa1523** | -0.1 | 0.3 |  |
| **Sa1528** | 0 | 0.3 |  |
| **Ccpa** | 0 | 0.3 |  |
| **Sa1570** | 0 | 0.3 |  |
| **Sa1582** | 0 | 0.3 |  |
| **Sa1613** | 0 | 0.3 |  |
| **Sa1703** | 0 | 0.3 |  |
| **Sa1709** | 0 | 0.3 |  |
| **Sa1727** | 0 | 0.3 |  |
| **Rplm** | 0 | 0.3 |  |
| **Rplr** | 0 | 0.3 |  |
| **Rpmc** | 0 | 0.3 |  |
| **Moeb** | 0 | 0.3 |  |
| **Sa2105** | 0 | 0.3 |  |
| **Sas084** | 0 | 0.3 |  |
| **Sas085** | 0 | 0.3 |  |
| **Sa2192** | 0 | 0.3 |  |
| **Sa2196** | 0 | 0.3 |  |
| **Sa2268** | 0 | 0.3 |  |
| **Sa2329** | 0 | 0.3 |  |
| **Pand** | 0 | 0.3 |  |
| **Sa2424** | 0 | 0.3 |  |
| **Bleo** | -0.5 | 0.3 |  |
| **Sa2148** | 0 | 0.3 |  |
| **Sa0370** | -0.1 | 0.3 |  |
| **Sa1308** | -0.1 | 0.3 |  |
| **Sa0036** | -0.1 | 0.3 |  |
| **Sa1388** | 0 | 0.3 |  |
| **Sa0254** | 0 | 0.3 |  |
| **Sa0295** | -0.1 | 0.3 |  |
| **Sa0466** | 0 | 0.3 |  |
| **Sa0681** | 0 | 0.3 |  |
| **Potd** | -0.1 | 0.3 |  |
| **Sa2248** | -0.1 | 0.3 |  |
| **Sa1792** | -0.1 | 0.3 |  |
| **Sen** | 0.1 | 0.3 |  |
| **Truncated(Repb)** | -0.5 | 0.3 |  |
| **Mura** | -0.1 | 0.3 |  |
| **Sa2416** | 0 | 0.3 |  |
| **Sa2300** | 0 | 0.3 |  |
| **Sa0316** | 0 | 0.3 |  |
| **Sa0970** | 0 | 0.3 |  |
| **Bfmbaa** | -0.1 | 0.3 |  |
| **Grpe** | 0 | 0.3 |  |
| **Sa1747** | -0.1 | 0.3 |  |
| **Sa2125** | 0 | 0.3 |  |
| **Hemn** | 0 | 0.3 |  |
| **Ftsz** | -0.1 | 0.3 |  |
| **Sa2440** | 0 | 0.3 |  |
| **Set/07** | 0 | 0.3 |  |
| **Sa2168** | 0 | 0.3 |  |
| **Sa2224** | 0 | 0.3 |  |
| **Yent1** | 0.1 | 0.3 |  |
| **Sa0142** | -0.1 | 0.3 |  |
| **Sa0513** | 0 | 0.3 |  |
| **Sa0574** | -0.1 | 0.3 |  |
| **Tgt** | 0 | 0.3 |  |
| **Sa0870** | 0 | 0.3 |  |
| **Sa0622** | -0.1 | 0.3 |  |
| **Sa0591** | 0 | 0.3 |  |
| **Sa0031** | -0.5 | 0.3 |  |
| **Sa0022** | -0.1 | 0.3 |  |
| **Sa1148** | 0 | 0.3 |  |
| **Sa0971** | -0.1 | 0.3 |  |
| **Trxb** | 0 | 0.3 |  |
| **Murd** | 0 | 0.3 |  |
| **Tig** | -0.1 | 0.3 |  |
| **Sa1812** | 0 | 0.3 |  |
| **Sa1928** | 0 | 0.3 |  |
| **Sa2393** | 0 | 0.3 |  |
| **Sa2171** | 0 | 0.2 |  |
| **Sa0540** | 0 | 0.2 |  |
| **Sa0610** | 0 | 0.2 |  |
| **Sa0675** | 0 | 0.2 |  |
| **Purd** | 0 | 0.2 |  |
| **Sas037** | 0 | 0.2 |  |
| **Phop** | 0 | 0.2 |  |
| **Sa1674** | 0 | 0.2 |  |
| **Sa2132** | 0 | 0.2 |  |
| **Sa0462** | 0 | 0.2 |  |
| **Dlta** | 0 | 0.2 |  |
| **Sa1256** | 0 | 0.2 |  |
| **Sa1476** | 0 | 0.2 |  |
| **Sa2353** | 0 | 0.2 |  |
| **Fhuc** | -0.1 | 0.2 |  |
| **Sa0092** | 0 | 0.2 |  |
| **Sa1813** | 0 | 0.2 |  |
| **Sa0079** | 0 | 0.2 |  |
| **Sas034** | 0 | 0.2 |  |
| **Glpd** | 0 | 0.2 |  |
| **Sa0875** | 0 | 0.2 |  |
| **Erma** | -0.7 | 0.2 |  |
| **Lpl2** | -0.1 | 0.2 |  |
| **Sa0551** | 0 | 0.2 |  |
| **Sa1192** | 0 | 0.2 |  |
| **Arg** | 0 | 0.2 |  |
| **Sa1307** | 0 | 0.2 |  |
| **Arcc** | 0 | 0.2 |  |
| **Sa0868** | -0.1 | 0.2 |  |
| **Potc** | 0 | 0.2 |  |
| **Sa1281** | 0 | 0.2 |  |
| **Purr** | 0 | 0.2 |  |
| **Sa0804** | 0 | 0.2 |  |
| **Sa1361** | 0 | 0.2 |  |
| **Sa1713** | 0 | 0.2 |  |
| **Sa0894** | -0.1 | 0.2 |  |
| **Sa1175** | 0 | 0.2 |  |
| **Fosb** | 0 | 0.2 |  |
| **Set/12** | 0 | 0.2 |  |
| **Sa1240** | -0.1 | 0.2 |  |
| **Sa0163** | 0 | 0.2 |  |
| **Mvak1** | 0 | 0.2 |  |
| **Tagb** | 0 | 0.2 |  |
| **Sa0832** | 0 | 0.2 |  |
| **Fabh** | 0 | 0.2 |  |
| **Purq** | 0 | 0.2 |  |
| **Sa0984** | 0 | 0.2 |  |
| **Sa1100** | -0.1 | 0.2 |  |
| **Asd** | 0 | 0.2 |  |
| **Sa1293** | 0 | 0.2 |  |
| **Sa1433** | 0 | 0.2 |  |
| **Sa1606** | -0.1 | 0.2 |  |
| **Putp** | 0 | 0.2 |  |
| **Sa1850** | 0 | 0.2 |  |
| **Sa1889** | 0 | 0.2 |  |
| **Sa1999** | 0 | 0.2 |  |
| **Nari** | 0 | 0.2 |  |
| **Sa2197** | 0 | 0.2 |  |
| **Sa2323** | -0.1 | 0.2 |  |
| **Sa2403** | 0 | 0.2 |  |
| **Isab** | -0.1 | 0.2 |  |
| **Gidb** | 0 | 0.2 |  |
| **Mvad** | 0 | 0.2 |  |
| **Sa0007** | 0 | 0.2 |  |
| **Sa0435** | 0 | 0.2 |  |
| **Sa0439** | 0 | 0.2 |  |
| **Rpll** | 0 | 0.2 |  |
| **Sa0557** | 0 | 0.2 |  |
| **Sa0560** | 0 | 0.2 |  |
| **Sa0563** | 0 | 0.2 |  |
| **Baca** | 0 | 0.2 |  |
| **Sa0641** | 0 | 0.2 |  |
| **Sa0665** | 0 | 0.2 |  |
| **Sa0697** | 0 | 0.2 |  |
| **Sa0724** | 0 | 0.2 |  |
| **Spsb** | 0 | 0.2 |  |
| **Sa1657** | 0 | 0.2 |  |
| **Sas054** | 0 | 0.2 |  |
| **Sa2242** | 0 | 0.2 |  |
| **Sa2451** | 0 | 0.2 |  |
| **Sa2472** | -0.1 | 0.2 |  |
| **Sa1667** | 0 | 0.2 |  |
| **Sa2250** | 0 | 0.2 |  |
| **Glpp** | 0 | 0.2 |  |
| **Sas057** | 0 | 0.2 |  |
| **Trpc** | 0 | 0.2 |  |
| **Aroe** | 0 | 0.2 |  |
| **Sa1156** | 0 | 0.2 |  |
| **Sa0940** | 0 | 0.2 |  |
| **Sa1163** | 0 | 0.2 |  |
| **Sa1636** | 0 | 0.2 |  |
| **Sa0081** | 0 | 0.2 |  |
| **Capn** | -0.1 | 0.2 |  |
| **Sa0320** | 0 | 0.2 |  |
| **Sa0356** | -0.1 | 0.2 |  |
| **Sas010** | 0 | 0.2 |  |
| **Sa0407** | 0 | 0.2 |  |
| **Sa0413** | 0 | 0.2 |  |
| **Sa0830** | 0 | 0.2 |  |
| **Fold** | 0 | 0.2 |  |
| **Sa0929** | 0 | 0.2 |  |
| **Sas030** | 0 | 0.2 |  |
| **Sa0941** | 0 | 0.2 |  |
| **Sa0988** | 0 | 0.2 |  |
| **Sa1019** | 0 | 0.2 |  |
| **Sa1021** | 0 | 0.2 |  |
| **Ftsl** | 0 | 0.2 |  |
| **Pyre** | 0 | 0.2 |  |
| **Hmrb** | 0 | 0.2 |  |
| **Sa1236** | -0.1 | 0.2 |  |
| **Sas046** | 0 | 0.2 |  |
| **Rpsu** | 0 | 0.2 |  |
| **Sa1453** | 0 | 0.2 |  |
| **Rplt** | 0 | 0.2 |  |
| **Murc** | 0 | 0.2 |  |
| **Sa1567** | 0 | 0.2 |  |
| **Sa1620** | 0 | 0.2 |  |
| **Rpmd** | 0 | 0.2 |  |
| **Rpln** | 0 | 0.2 |  |
| **Sa2110** | 0 | 0.2 |  |
| **Sa2179** | 0 | 0.2 |  |
| **Sa2359** | 0 | 0.2 |  |
| **Sa2401** | 0 | 0.2 |  |
| **Sa0103** | 0 | 0.2 |  |
| **Clpq** | 0 | 0.2 |  |
| **Cody** | 0 | 0.2 |  |
| **Sa1124** | 0 | 0.2 |  |
| **Sa1335** | 0 | 0.2 |  |
| **Rplo** | 0 | 0.2 |  |
| **Sa2170** | 0 | 0.2 |  |
| **Nasf** | 0 | 0.2 |  |
| **Sa2223** | 0 | 0.2 |  |
| **Sa0170** | 0 | 0.2 |  |
| **Sa0350** | 0 | 0.2 |  |
| **Sa0928** | -0.1 | 0.2 |  |
| **Sa1278** | 0 | 0.2 |  |
| **Sa2081** | -0.1 | 0.2 |  |
| **Biod** | 0 | 0.2 |  |
| **Sa1748** | 0 | 0.2 |  |
| **Sa1536** | 0 | 0.2 |  |
| **Sa2307** | 0 | 0.2 |  |
| **Sa1064** | 0 | 0.2 |  |
| **Infc** | 0 | 0.2 |  |
| **Sa1559** | 0 | 0.2 |  |
| **Sa0216** | 0 | 0.2 |  |
| **Sa0748** | 0 | 0.2 |  |
| **Ilvd** | 0 | 0.2 |  |
| **Sa2244** | 0 | 0.2 |  |
| **Sa0285** | 0 | 0.2 |  |
| **Gap** | 0 | 0.2 |  |
| **Sas038** | 0 | 0.2 |  |
| **Sa1265** | 0 | 0.2 |  |
| **Hemx** | 0 | 0.2 |  |
| **Sa2377** | 0 | 0.2 |  |
| **Sa0652** | 0 | 0.2 |  |
| **Sa0771** | 0 | 0.2 |  |
| **Odhb** | 0 | 0.2 |  |
| **Sa1675** | 0 | 0.2 |  |
| **Sa1449** | -0.1 | 0.2 |  |
| **Sas023** | 0 | 0.2 |  |
| **Sa1325** | 0 | 0.2 |  |
| **Seo** | 0 | 0.2 |  |
| **Sa1782** | -0.3 | 0.2 |  |
| **Bsaa** | -0.1 | 0.2 |  |
| **Sa1161** | 0 | 0.2 |  |
| **Capk** | 0 | 0.2 |  |
| **Sa0139** | 0 | 0.2 |  |
| **Gudb** | 0 | 0.2 |  |
| **Sa0827** | -0.1 | 0.2 |  |
| **Sa0974** | 0 | 0.2 |  |
| **Srtb** | 0 | 0.2 |  |
| **Arls** | 0 | 0.2 |  |
| **Sa1289** | 0 | 0.2 |  |
| **Sa1343** | 0 | 0.2 |  |
| **Sa1395** | 0 | 0.2 |  |
| **Gbsa** | 0 | 0.2 |  |
| **Sa1179** | 0 | 0.2 |  |
| **Sa2191** | 0 | 0.2 |  |
| **Pflb** | 0 | 0.2 |  |
| **Sa0248** | -0.1 | 0.2 |  |
| **Sa0485** | 0 | 0.2 |  |
| **Sa1016** | 0 | 0.2 |  |
| **Sa2320** | 0 | 0.2 |  |
| **Sa1595** | 0 | 0.2 |  |
| **Sa1744** | 0 | 0.2 |  |
| **Sa2094** | 0 | 0.2 |  |
| **Sa2056** | 0 | 0.2 |  |
| **Soda** | 0 | 0.2 |  |
| **Sa0824** | 0 | 0.2 |  |
| **Sa1393** | 0 | 0.2 |  |
| **Sa0315** | 0 | 0.2 |  |
| **Sa0711** | 0 | 0.2 |  |
| **Tpi** | 0 | 0.2 |  |
| **Malr** | 0 | 0.2 |  |
| **Sa2466** | 0 | 0.2 |  |
| **Sa2490** | 0 | 0.2 |  |
| **Sa0129** | 0 | 0.2 |  |
| **Sa0637** | 0 | 0.2 |  |
| **Dnai** | 0 | 0.2 |  |
| **Sa1851** | 0 | 0.2 |  |
| **Czrb** | -0.1 | 0.2 |  |
| **Lacf** | -0.1 | 0.2 |  |
| **Uref** | 0 | 0.2 |  |
| **Sa2127** | 0 | 0.2 |  |
| **Sa0426** | -0.1 | 0.2 |  |
| **Sa0541** | 0 | 0.2 |  |
| **Sa0684** | 0 | 0.2 |  |
| **Opp-2C** | 0 | 0.2 |  |
| **Sa2001** | -0.1 | 0.2 |  |
| **Sas076** | 0 | 0.2 |  |
| **Sa0537** | 0 | 0.2 |  |
| **Sa0649** | 0 | 0.2 |  |
| **Fabd** | -0.1 | 0.2 |  |
| **Sa2149** | 0 | 0.2 |  |
| **Sa1373** | 0 | 0.2 |  |
| **Pyrab** | -0.1 | 0.2 |  |
| **Sas019** | 0.1 | 0.1 |  |
| **Sa2169** | 0 | 0.1 |  |
| **Agra** | 0 | 0.1 |  |
| **Sa0126** | -0.2 | 0.1 |  |
| **Sbne** | -0.1 | 0.1 |  |
| **Lpl5** | 0 | 0.1 |  |
| **Isdd** | 0 | 0.1 |  |
| **Sa1093** | 0 | 0.1 |  |
| **Sa1333** | 0 | 0.1 |  |
| **Sa1868** | -0.1 | 0.1 |  |
| **Sa1946** | 0 | 0.1 |  |
| **Sa1341** | 0 | 0.1 |  |
| **Sa1791** | -0.1 | 0.1 |  |
| **Sa1159** | 0 | 0.1 |  |
| **Sa2444** | -0.1 | 0.1 |  |
| **Sa2277** | -0.1 | 0.1 |  |
| **Sa0395** | 0 | 0.1 |  |
| **Sa0409** | 0 | 0.1 |  |
| **Sa0424** | 0 | 0.1 |  |
| **Sa0846** | 0 | 0.1 |  |
| **Sa0861** | 0 | 0.1 |  |
| **Sa0862** | 0 | 0.1 |  |
| **Sa1383** | 0 | 0.1 |  |
| **Sa1501** | 0 | 0.1 |  |
| **Ribb** | 0 | 0.1 |  |
| **Sa1707** | 0 | 0.1 |  |
| **Sa1739** | 0 | 0.1 |  |
| **Moab** | 0 | 0.1 |  |
| **Sa2160** | 0 | 0.1 |  |
| **Sa2174** | 0 | 0.1 |  |
| **Sa2210** | 0 | 0.1 |  |
| **Sa2280** | 0 | 0.1 |  |
| **Sa2281** | 0 | 0.1 |  |
| **Gntr** | 0 | 0.1 |  |
| **Sa2322** | 0 | 0.1 |  |
| **Sa2328** | -0.1 | 0.1 |  |
| **Sa2363** | -0.1 | 0.1 |  |
| **Sa2372** | 0 | 0.1 |  |
| **Sa2399** | -0.1 | 0.1 |  |
| **Sa0558** | 0 | 0.1 |  |
| **Pept** | 0 | 0.1 |  |
| **Sa1486** | 0 | 0.1 |  |
| **Sa0770** | 0 | 0.1 |  |
| **Ctab** | 0 | 0.1 |  |
| **Sa1145** | 0 | 0.1 |  |
| **Sa1891** | 0 | 0.1 |  |
| **Sa2439** | -0.1 | 0.1 |  |
| **Lytm** | -0.1 | 0.1 |  |
| **Glpt** | 0 | 0.1 |  |
| **Pria** | -0.1 | 0.1 |  |
| **Sa1530** | 0 | 0.1 |  |
| **Atpg** | 0 | 0.1 |  |
| **Sa2203** | 0 | 0.1 |  |
| **Sa2249** | -0.1 | 0.1 |  |
| **Sa0136** | 0 | 0.1 |  |
| **Sa0437** | 0 | 0.1 |  |
| **Sa1223** | 0 | 0.1 |  |
| **Sa1420** | 0 | 0.1 |  |
| **Sa1455** | 0 | 0.1 |  |
| **Sa1535** | 0 | 0.1 |  |
| **Sa2325** | 0 | 0.1 |  |
| **Caph** | 0 | 0.1 |  |
| **Sa0175** | 0 | 0.1 |  |
| **Sa0338** | 0 | 0.1 |  |
| **Sa0892** | 0 | 0.1 |  |
| **Sspc** | 0 | 0.1 |  |
| **Sa0919** | 0 | 0.1 |  |
| **Purn** | 0 | 0.1 |  |
| **Sa0989** | 0 | 0.1 |  |
| **Sa1032** | 0 | 0.1 |  |
| **Gmk** | 0 | 0.1 |  |
| **Rpsp** | -0.1 | 0.1 |  |
| **Smba** | 0 | 0.1 |  |
| **Sa1332** | 0 | 0.1 |  |
| **Sa1376** | 0 | 0.1 |  |
| **Ruva** | 0 | 0.1 |  |
| **Sa1540** | 0 | 0.1 |  |
| **Sa2190** | 0 | 0.1 |  |
| **Sa2324** | 0 | 0.1 |  |
| **Sa2352** | 0 | 0.1 |  |
| **Sa1066** | 0 | 0.1 |  |
| **Rpsk** | 0 | 0.1 |  |
| **Nana** | 0 | 0.1 |  |
| **Rpsr** | 0 | 0.1 |  |
| **Sa0878** | 0 | 0.1 |  |
| **Grea** | 0 | 0.1 |  |
| **Sa1591** | 0 | 0.1 |  |
| **Trua** | 0 | 0.1 |  |
| **Rpld** | 0 | 0.1 |  |
| **Uree** | -0.1 | 0.1 |  |
| **Sa2139** | -0.1 | 0.1 |  |
| **Sa2306** | 0 | 0.1 |  |
| **Sa2330** | 0 | 0.1 |  |
| **Sa2346** | -0.1 | 0.1 |  |
| **Sbnd** | 0 | 0.1 |  |
| **Sa0229** | 0 | 0.1 |  |
| **Ccrb** | -0.7 | 0.1 |  |
| **Sa0312** | -0.1 | 0.1 |  |
| **Sa1635** | 0 | 0.1 |  |
| **Sa1063** | -0.1 | 0.1 |  |
| **Sa0508** | 0 | 0.1 |  |
| **Femb** | 0 | 0.1 |  |
| **Fdh** | -0.1 | 0.1 |  |
| **Sigb** | 0 | 0.1 |  |
| **Sa1958** | 0 | 0.1 |  |
| **Ssaa** | 0 | 0.1 |  |
| **Sa2470** | 0 | 0.1 |  |
| **Sa0047** | -0.4 | 0.1 |  |
| **Guab** | -0.1 | 0.1 |  |
| **Sa0668** | -0.1 | 0.1 |  |
| **Isde** | 0 | 0.1 |  |
| **Trmd** | -0.1 | 0.1 |  |
| **Sa1359** | -0.1 | 0.1 |  |
| **Mene** | -0.1 | 0.1 |  |
| **Sa1740** | 0 | 0.1 |  |
| **Mqo2** | 0 | 0.1 |  |
| **Sa2481** | 0 | 0.1 |  |
| **Gcad** | -0.1 | 0.1 |  |
| **Lexa** | 0 | 0.1 |  |
| **Sa1477** | 0 | 0.1 |  |
| **Sa2141** | 0 | 0.1 |  |
| **Sa2222** | -0.1 | 0.1 |  |
| **Sa2239** | -0.2 | 0.1 |  |
| **Gida** | 0 | 0.1 |  |
| **Truncated(Hlb)** | 0 | 0.1 |  |
| **Sdre** | 0 | 0.1 |  |
| **Sa1215** | 0 | 0.1 |  |
| **Sa1371** | 0 | 0.1 |  |
| **Sa0528** | -0.2 | 0.1 |  |
| **Sa1011** | 0 | 0.1 |  |
| **Sa0211** | 0 | 0.1 |  |
| **Sa0228** | 0 | 0.1 |  |
| **Sa0954** | 0 | 0.1 |  |
| **Cudt** | -0.1 | 0.1 |  |
| **Sa0858** | 0 | 0.1 |  |
| **Secy** | 0 | 0.1 |  |
| **Sa0565** | 0 | 0.1 |  |
| **Sa1497** | 0 | 0.1 |  |
| **Sa2162** | 0 | 0.1 |  |
| **Sa0589** | 0 | 0.1 |  |
| **Sa0884** | 0 | 0.1 |  |
| **Mtld** | -0.1 | 0.1 |  |
| **Capf** | -0.1 | 0.1 |  |
| **Rbsd** | 0 | 0.1 |  |
| **Sa0294** | 0 | 0.1 |  |
| **Sa0317** | 0 | 0.1 |  |
| **Sa0380** | -0.1 | 0.1 |  |
| **Sa0422** | -0.1 | 0.1 |  |
| **Recr** | 0 | 0.1 |  |
| **Sa0441** | 0 | 0.1 |  |
| **Sa0490** | 0 | 0.1 |  |
| **Sa0601** | 0 | 0.1 |  |
| **Sa0625** | 0 | 0.1 |  |
| **Sa0643** | 0 | 0.1 |  |
| **Ssrp** | -0.1 | 0.1 |  |
| **Purh** | -0.1 | 0.1 |  |
| **Sas031** | 0 | 0.1 |  |
| **Muri** | 0 | 0.1 |  |
| **Sa1031** | 0 | 0.1 |  |
| **Sa1241** | 0 | 0.1 |  |
| **Dfra** | 0 | 0.1 |  |
| **Sa1364** | 0 | 0.1 |  |
| **Obg** | 0 | 0.1 |  |
| **Heme** | 0 | 0.1 |  |
| **Sa1697** | 0 | 0.1 |  |
| **Sa1897** | 0 | 0.1 |  |
| **Sa2098** | -0.2 | 0.1 |  |
| **Sa2115** | 0 | 0.1 |  |
| **Sa2189** | -0.1 | 0.1 |  |
| **Isaa** | 0 | 0.1 |  |
| **Luke** | 0 | 0.1 |  |
| **Sa0030** | -0.6 | 0.1 |  |
| **Sa0243** | -0.1 | 0.1 |  |
| **Tnpb** | -0.3 | 0.1 |  |
| **Sa2052** | 0 | 0.1 |  |
| **Pre** | -0.6 | 0.1 |  |
| **Aadd** | 0 | 0.1 |  |
| **Sa1625** | -0.1 | 0.1 |  |
| **Sa1639** | -0.1 | 0.1 |  |
| **Sa0080** | -0.1 | 0.1 |  |
| **Sa0544** | 0 | 0.1 |  |
| **Sa0609** | -0.1 | 0.1 |  |
| **Recq** | 0 | 0.1 |  |
| **Sa0694** | 0 | 0.1 |  |
| **Sa0710** | 0 | 0.1 |  |
| **Eno** | 0 | 0.1 |  |
| **Sa0802** | 0 | 0.1 |  |
| **Oppd** | 0 | 0.1 |  |
| **Xerc** | 0 | 0.1 |  |
| **Sa1478** | 0 | 0.1 |  |
| **Folc** | -0.1 | 0.1 |  |
| **Sa1668** | 0 | 0.1 |  |
| **Pdp** | -0.2 | 0.1 |  |
| **Msmx** | 0 | 0.1 |  |
| **Mtla** | 0 | 0.1 |  |
| **Tnpa** | -0.3 | 0.1 |  |
| **Sa0767** | -0.4 | 0.1 |  |
| **Sa1479** | -0.4 | 0.1 |  |
| **Sa1950** | -0.4 | 0.1 |  |
| **Sa2383** | -0.4 | 0.1 |  |
| **Sa0753** | 0 | 0.1 |  |
| **Potb** | 0 | 0.1 |  |
| **Hema** | 0 | 0.1 |  |
| **Sas041** | 0 | 0.1 |  |
| **Gpsa** | 0 | 0.1 |  |
| **Sa2155** | 0 | 0.1 |  |
| **Rpmb** | 0 | 0.1 |  |
| **Sa1549** | 0 | 0.1 |  |
| **Rpss** | 0 | 0.1 |  |
| **Sa2166** | 0 | 0.1 |  |
| **Sa2218** | 0 | 0.1 |  |
| **Sa2370** | 0 | 0.1 |  |
| **Sa0330** | 0 | 0.1 |  |
| **Metb** | 0 | 0.1 |  |
| **Sas032** | 0 | 0.1 |  |
| **Sa1053** | 0 | 0.1 |  |
| **Sa1327** | -0.1 | 0.1 |  |
| **Sa1538** | 0 | 0.1 |  |
| **Sa2205** | 0 | 0.1 |  |
| **Sa2313** | 0 | 0.1 |  |
| **Sa2318** | 0 | 0.1 |  |
| **Sa2412** | 0 | 0.1 |  |
| **Sa2422** | 0 | 0.1 |  |
| **Vrar** | 0 | 0.1 |  |
| **Sa0960** | 0 | 0.1 |  |
| **Sa0999** | 0 | 0.1 |  |
| **Pcrb** | -0.1 | 0.1 |  |
| **Rplv** | 0 | 0.1 |  |
| **Sa1078** | 0 | 0.1 |  |
| **Bex** | 0 | 0.1 |  |
| **Lpl1** | 0 | 0.1 |  |
| **Sa2315** | -0.2 | 0.1 |  |
| **Sa1585** | 0 | 0.1 |  |
| **Ndhf** | 0 | 0.1 |  |
| **Sa0599** | 0 | 0.1 |  |
| **Msra** | 0 | 0.1 |  |
| **Sa1385** | 0 | 0.1 |  |
| **Lyss** | -0.1 | 0.1 |  |
| **Pota** | 0 | 0.1 |  |
| **Sa0958** | 0 | 0.1 |  |
| **Sa1380** | 0 | 0.1 |  |
| **Lyts** | -0.1 | 0.1 |  |
| **Sa0012** | -0.1 | 0.1 |  |
| **Sa0260** | -0.1 | 0.1 |  |
| **Sa0281** | 0 | 0.1 |  |
| **Sa0801** | 0 | 0.1 |  |
| **Opp-2F** | 0 | 0.1 |  |
| **Gercc** | 0 | 0.1 |  |
| **Ispa** | 0 | 0.1 |  |
| **Sa1529** | 0 | 0.1 |  |
| **Sa1665** | 0 | 0.1 |  |
| **Sa1972** | -0.2 | 0.1 |  |
| **Sa2374** | -0.2 | 0.1 |  |
| **Arcd** | 0 | 0.1 |  |
| **Nade** | 0 | 0.1 |  |
| **Sa0319** | 0 | 0.1 |  |
| **Frr** | 0 | 0.1 |  |
| **Sa0181** | 0 | 0.1 |  |
| **Sa0328** | 0 | 0.1 |  |
| **Sa0425** | 0 | 0.1 |  |
| **Sa0829** | 0 | 0.1 |  |
| **Sa0908** | 0 | 0.1 |  |
| **Qoxc** | 0 | 0.1 |  |
| **Sa0973** | 0 | 0.1 |  |
| **Sa0998** | 0 | 0.1 |  |
| **Rpls** | 0 | 0.1 |  |
| **Sa1111** | 0 | 0.1 |  |
| **Sa1329** | 0 | 0.1 |  |
| **Sa1330** | 0 | 0.1 |  |
| **Cdd** | 0 | 0.1 |  |
| **Sa1399** | 0 | 0.1 |  |
| **Sa1422** | 0 | 0.1 |  |
| **Rpma** | 0 | 0.1 |  |
| **Sas051** | 0 | 0.1 |  |
| **Rpmi** | 0 | 0.1 |  |
| **Sa1509** | 0 | 0.1 |  |
| **Pfk** | 0 | 0.1 |  |
| **Rple** | 0 | 0.1 |  |
| **Rplp** | 0 | 0.1 |  |
| **Sbnh** | 0 | 0.1 |  |
| **Sa0307** | 0 | 0.1 |  |
| **Sa0814** | -0.1 | 0.1 |  |
| **Sa1068** | 0 | 0.1 |  |
| **Rplw** | 0 | 0.1 |  |
| **Sas086** | 0 | 0.1 |  |
| **Sa2202** | 0 | 0.1 |  |
| **Sa2342** | 0 | 0.1 |  |
| **Sa2419** | 0 | 0.1 |  |
| **Sa0337** | 0 | 0.1 |  |
| **Sa2414** | 0 | 0.1 |  |
| **Sa1318** | 0 | 0.1 |  |
| **Sa0788** | 0 | 0 |  |
| **Gltx** | -0.1 | 0 |  |
| **Sa0872** | 0 | 0 |  |
| **Pnpa** | -0.1 | 0 |  |
| **Sa1122** | 0 | 0 |  |
| **Sa1291** | 0 | 0 |  |
| **Sa2002** | 0 | 0 |  |
| **Sa2257** | 0 | 0 |  |
| **Sa2396** | 0 | 0 |  |
| **Sa0186** | 0 | 0 |  |
| **Sa1010** | 0 | 0 |  |
| **Mscl** | 0 | 0 |  |
| **Sa2015** | 0 | 0 |  |
| **Sa1176** | 0 | 0 |  |
| **Sa1366** | 0 | 0 |  |
| **Mutl** | 0 | 0 |  |
| **Glct** | 0 | 0 |  |
| **Sas040** | 0 | 0 |  |
| **Sa2273** | 0 | 0 |  |
| **Sa1363** | -0.1 | 0 |  |
| **Sa2366** | -0.1 | 0 |  |
| **Sa0137** | -0.1 | 0 |  |
| **Sa0646** | 0 | 0 |  |
| **Glpq** | -0.1 | 0 |  |
| **Sa0972** | 0 | 0 |  |
| **Sdhb** | 0 | 0 |  |
| **Sucd** | 0 | 0 |  |
| **Upps** | 0 | 0 |  |
| **Sa1337** | 0 | 0 |  |
| **Ahrc** | 0 | 0 |  |
| **Dnaj** | 0 | 0 |  |
| **Pfs** | 0 | 0 |  |
| **Hit** | 0 | 0 |  |
| **Sa1729** | 0 | 0 |  |
| **Ilvc** | -0.1 | 0 |  |
| **Sa1885** | 0 | 0 |  |
| **Ddla** | 0 | 0 |  |
| **Rplq** | 0 | 0 |  |
| **Sa2256** | 0 | 0 |  |
| **Sa2262** | -0.1 | 0 |  |
| **Sa2297** | 0 | 0 |  |
| **Pfla** | 0 | 0 |  |
| **Sa1745** | 0 | 0 |  |
| **Sa2452** | 0 | 0 |  |
| **Sa1725** | 0 | 0 |  |
| **Bgla** | -0.1 | 0 |  |
| **Uhpt** | 0 | 0 |  |
| **Sa0408** | 0 | 0 |  |
| **Sa1040** | 0 | 0 |  |
| **Rnhb** | 0 | 0 |  |
| **Succ** | 0 | 0 |  |
| **Acca** | 0 | 0 |  |
| **Sa1596** | 0 | 0 |  |
| **Sa2005** | -0.1 | 0 |  |
| **Sa2049** | 0 | 0 |  |
| **Sa2229** | 0 | 0 |  |
| **Sa2243** | 0 | 0 |  |
| **Sa0269** | -0.1 | 0 |  |
| **Sarh2** | 0 | 0 |  |
| **Sa0288** | 0 | 0 |  |
| **Recg** | -0.1 | 0 |  |
| **Sa1598** | 0 | 0 |  |
| **Recf** | 0 | 0 |  |
| **Vicr** | 0 | 0 |  |
| **Lcte** | 0 | 0 |  |
| **Sa0678** | 0 | 0 |  |
| **Sa0789** | -0.1 | 0 |  |
| **Dltb** | 0 | 0 |  |
| **Sa1018** | -0.1 | 0 |  |
| **Gid** | 0 | 0 |  |
| **Aroc** | 0 | 0 |  |
| **Sa1367** | 0 | 0 |  |
| **Sa1425** | 0 | 0 |  |
| **Vga** | 0 | 0 |  |
| **Rplb** | 0 | 0 |  |
| **Rplc** | 0 | 0 |  |
| **Lytr** | 0 | 0 |  |
| **Sa0443** | 0 | 0 |  |
| **Vraf** | 0 | 0 |  |
| **Sa0190** | 0 | 0 |  |
| **Sa0261** | 0 | 0 |  |
| **Sa0291** | 0 | 0 |  |
| **Sa0301** | 0 | 0 |  |
| **Sa0348** | -0.1 | 0 |  |
| **Sa0552** | 0 | 0 |  |
| **Sa0566** | 0 | 0 |  |
| **Saer** | 0 | 0 |  |
| **Fab** | 0 | 0 |  |
| **Mure** | -0.1 | 0 |  |
| **Plsx** | -0.1 | 0 |  |
| **Sa2112** | 0 | 0 |  |
| **Huti** | 0 | 0 |  |
| **Sa2150** | -0.1 | 0 |  |
| **Sa2233** | 0 | 0 |  |
| **Sa0087** | 0 | 0 |  |
| **Sa0090** | 0 | 0 |  |
| **Sa0165** | 0 | 0 |  |
| **Sa0340** | 0 | 0 |  |
| **Sa0874** | 0 | 0 |  |
| **Pdf1** | 0 | 0 |  |
| **Sa0961** | 0 | 0 |  |
| **Sdhc** | 0 | 0 |  |
| **Sdha** | 0 | 0 |  |
| **Sa1017** | 0 | 0 |  |
| **Sa1035** | 0 | 0 |  |
| **Pyrf** | 0 | 0 |  |
| **Sa1378** | -0.1 | 0 |  |
| **Sa1402** | 0 | 0 |  |
| **Sa1407** | 0 | 0 |  |
| **Sa1421** | 0 | 0 |  |
| **Sa1469** | 0 | 0 |  |
| **Sa1594** | 0 | 0 |  |
| **Sa1735** | 0 | 0 |  |
| **Rplf** | 0 | 0 |  |
| **Narq** | 0 | 0 |  |
| **Bioa** | -0.2 | 0 |  |
| **Sa2267** | 0 | 0 |  |
| **Sa2417** | 0 | 0 |  |
| **Sa2438** | 0 | 0 |  |
